# An Examination of Whether Mindfulness Can Predict the Relationship Between Objective and Subjective Attitudinal Ambivalence

**DOI:** 10.3389/fpsyg.2019.00854

**Published:** 2019-04-24

**Authors:** Jennifer Weng, Kenneth G. DeMarree

**Affiliations:** Department of Psychology, University at Buffalo, The State University of New York, Buffalo, NY, United States

**Keywords:** attitudes, ambivalence, mindfulness, metacognition, acceptance

## Abstract

Ambivalence is a mixed reaction toward an attitudinal object. Ambivalence is often viewed as aversive and people are motivated to reduce it. However, the presence of both strong positive and negative attitudes toward an object (objective ambivalence; OA) does not always lead to consciously experienced conflicted and torn feelings (subjective ambivalence; SA) or psychological discomfort. We hypothesized that the way people think about their inner experience can affect whether ambivalent attitudes lead to increased conflicted feelings. In five studies, we examined whether mindfulness predicts the relationship between objective and subjective ambivalence. We predicted that the acceptance aspect of mindfulness would attenuate the relationship between OA and SA, based on the idea that acceptance makes people more tolerant and less judgmental toward their inner states in general (and OA in particular). Although some findings across five studies were consistent with the prediction showing that acceptance attenuated the OA–SA relationship, other findings were not and even showed that acceptance strengthened the OA–SA relationship. A meta-analysis of the interaction effect across all studies failed to find support for predictions (*r* = -0.036 and 95% CI [-0.087; 0.022]). We discuss possible reasons for these mixed findings, and the implications of these studies.

## Introduction

Ambivalence is a mixed reaction toward an attitude object (Kaplan, 1972). People can have ambivalent attitudes toward anything from consumer products to political or social issues to other people or groups of people (e.g., celebrities). Understanding ambivalence is important because ambivalent attitudes are usually less predictive of behavior and more prone to persuasion than are unambivalent attitudes (e.g., Armitage and Conner, 2000; Bell and Esses, 2002; for an exception, see Sawicki et al., 2013). There is an important distinction between two types of ambivalence: objective ambivalence (OA) and subjective ambivalence (SA) (Thompson et al., 1995; Priester and Petty, 1996). Objective ambivalence is the presence of both strong positive and negative evaluations toward an object. Subjective ambivalence is the conscious and direct *experience* of evaluative conflict. Attitudinal ambivalence, especially SA, is viewed as aversive and people are motivated to reduce it (van Harreveld et al., 2009). Further, SA has been conceptually and empirically implicated as the key driving force behind many effects of ambivalence [e.g., biased information processing (Nordgren et al., 2006); attitude-behavior correspondence, and the desire to reduce attitudinal conflict (DeMarree et al., 2014); for a review, see van Harreveld et al., 2015]. Consequently, it is important to understand when or for whom OA will lead to SA.

In general, people who have higher objective ambivalence, having mixed reactions toward an attitude object, tend to experience higher subjective ambivalence, having conflicted, torn, and uncomfortable feelings. Previous research showed that these two types of ambivalence are positively correlated around *r* = 0.4 to 0.5, and in experimental studies which used novel topics (e.g., mixed positive and negative traits about a target person) to manipulate OA, OA consistently affects SA (e.g., Priester and Petty, 1996). However, although the OA–SA relationship is generally a medium to large correlation, it is far from strong enough to view them as interchangeable, and previous research also identified some attitude-specific factors and person-specific factors or individual differences that moderate the relationship. For example, attitude-specific factors such as the simultaneous accessibility of both reactions (Newby-Clark et al., 2002) or similar levels of certainty toward each reaction (DeMarree et al., 2015) predict a stronger relationship between OA and SA. Person-specific factors, such as someone’s tolerance of cognitive inconsistency can also moderate the effect, with greater preference for consistency predicting a stronger relationship between OA and SA (Newby-Clark et al., 2002). In the present research, we examined another individual difference that may moderate the relationship between OA and SA. We predicted that the specific metacognitive perspectives people adopt with respect to their internal states in general, and their ambivalent attitudes in particular, would affect the subjective ambivalence they experience. That is, the metacognitive relationships people have with their thoughts may play an important role in the experience of ambivalence. As described in detail below, we conceptualize mindfulness as an individual difference in people’s metacognitive perspective on their thoughts, and in this research, we examined whether the metacognitive variable of mindfulness moderates the relationship between OA and SA.

Mindfulness is defined as “paying attention in a particular way: on purpose, in the present moment, and non-judgmentally” (Kabat-Zinn, 1994, p. 4). Although there are many mindfulness-related constructs discussed in the literature, we focus on two components that have often been identified as core to mindfulness: present-moment awareness and non-judgmental acceptance (e.g., Bishop et al., 2004; Cardaciotto et al., 2008; Coffey et al., 2010; Cameron and Fredrickson, 2015; Lindsay and Creswell, 2017). Present-moment awareness is the sustained attention toward one’s current thoughts, emotions, physical sensations, and environment. Non-judgmental acceptance is an open, curious, and non-judgmental attitude toward these experiences. When applied to one’s mental contents, both present-moment awareness and acceptance are metacognitive in nature, because they reflect thoughts about thoughts (for reviews of metacognition, see Dunlosky and Metcalfe, 2009; Briñol and DeMarree, 2012). That is, changes in awareness and acceptance do not necessarily reflect a shift in the content of one’s primary thoughts and feelings, but reflect a shift in one’s awareness of and relationship to these thoughts. Although both awareness and acceptance may relate to previously considered types of metacognition (e.g., metacognitive monitoring; see Dunlosky and Metcalfe, 2009), the acceptance component of mindfulness may be particularly important because acceptance has been implicated in facilitating effective emotion-regulation (e.g., Rahl et al., 2017).

It is worth noting that some mindfulness conceptualizations map directly onto the awareness versus acceptance dimensions, but others do not. The Philadelphia Mindfulness Scale, for example, attempts to directly assess these two concepts (Cardaciotto et al., 2008). Some work has attempted to manipulate the awareness and acceptance aspects of mindfulness separately through long-term training (e.g., Rahl et al., 2017). And still other work has argued for these dimensions based on a combination of conceptual and empirical analyses (e.g., Coffey et al., 2010). In other approaches, hints of these concepts can be found, even though they are not explicitly labeled as such. For example, the Five Facet Mindfulness Scale (Baer et al., 2006) has one facet that reflects present moment awareness, which is typically labeled “observing” and multiple facets that appear related to acceptance aspects of mindfulness, including non-judging and non-reactivity, though other dimensions assessed do not fit as neatly into the acceptance and awareness conceptualization (labeling and acting with awareness). In addition, Bernstein et al. (2015) identify meta-awareness, disidentification from internal experience, and reduced reactivity to thought content as core aspects of the mindfulness-related concept of decentering (see also Naragon-Gainey and DeMarree, 2017b). Of these, meta-awareness maps onto our conceptualization of awareness, and the latter two concepts appear to be related to acceptance and its consequences.

Initial work on mindfulness and attitudes was based on the idea that mindfulness can reduce inconsistencies within attitudes or between attitudes and behaviors. For instance, in one study, people with higher mindfulness showed greater congruence between their intentions and behaviors (Chatzisarantis and Hagger, 2007). This may be because mindfulness increases people’s awareness and acceptance of the intention they have, so they are more likely to fully endorse and act on their intention. Similarly, meditation also increases the congruence between explicit and implicit self-esteem (i.e., people’s self-attitude; Koole et al., 2009).

Building off of the idea that mindfulness can reduce inconsistencies (see also Crane et al., 2008), Haddock et al. (2017) showed that as mindfulness increased, people reported being more comfortable holding ambivalent attitudes and reported experiencing ambivalent attitudes less often. They also showed that mindfulness predicted less OA and SA across a number of topics (see also Dummel, 2018). However, our focus differs from this past work. Specifically, our primary focus is on the question of whether mindfulness might predict the *relationship between* OA and SA.

There are at least two related reasons why mindfulness in general, and acceptance in particular, might attenuate the link between OA and SA. The first reason is that mindfulness might reduce automatic, habitual, or default responding (for a review, see Kang et al., 2013). Notably, past work has found that mindfulness-related concepts predict reduced impact of traits (e.g., Feltman et al., 2009), habits (e.g., Wenk-Sormaz, 2005), and impulses (e.g., Papies et al., 2012). If we assume that the automatic or default response of OA is to experience SA, then mindfulness might decouple the link between OA and SA because of this de-automatization.

The second reason is based on the notion that mindfulness helps with the regulation of emotions, pain, and other aversive experiences, and OA is a potentially aversive experience (i.e., it can lead to SA). A substantial body of evidence suggests that mindfulness can make typically negative experiences less bothersome. For example, previous research showed that mindfulness was positively correlated with the ability to deal effectively with negative emotions (Coffey et al., 2010) and increased participants’ willingness to tolerate uncomfortable emotions and experiences (Levitt et al., 2004; Arch and Craske, 2006). Additionally, mindfulness-related concepts (e.g., Kiken and Shook, 2012; Naragon-Gainey and DeMarree, 2017a) and acceptance in particular (Campbell-Sills et al., 2006) predict reduced emotional responses or distress in response to negative experiences. So, it seems plausible that mindfulness could help people deal with and tolerate the ambivalent attitudes they have, and they may be less bothered by the presence of conflict. Critically, effects of mindfulness on emotion regulation processes (Teper and Inzlicht, 2013; Lindsay and Creswell, 2015) are generally thought to be due to acceptance aspects of mindfulness (Lindsay and Creswell, 2017).

In this research, we conceptualize mindfulness as two components, awareness and acceptance. Our predictions primarily focused on acceptance facet, because accepting, non-judgmental, and non-reactive responses to one’s inner experiences should facilitate the processes described above. We predicted that acceptance would attenuate the relationship between OA and SA, because acceptance should make people more tolerant and less judgmental toward their conflicting reactions, which could then lead them to feel less SA and less psychological discomfort and associated negative affect.

We did not make explicit predictions for awareness, as multiple predictions seemed plausible. On the one hand, awareness of both conflicting reactions could increase the relationship of OA with SA, consistent with past work on simultaneous accessibility of conflicting reactions (Newby-Clark et al., 2002). On the other hand, because awareness – especially if this awareness is psychologically detached – might be an important precursor or component of acceptance, it could also attenuate the relationship between OA and SA for similar reasons to those hypothesized for acceptance.

We tested these ideas across five studies: two correlational studies that examined these relationships across people’s pre-existing attitudes and three experimental studies that attempted to manipulate ambivalence. In the methods of each study, we report all measures and all manipulations, either in the main text or in a footnote. Any materials not described in detail in the main text are described in detail in the Supplementary Materials. All studies were approved by the University at Buffalo Institutional Review Board, and all subjects gave informed consent to participate in the studies.

## Study 1

The goal of Study 1 is to test whether mindfulness moderates the relationship between objective and subjective ambivalence in existing attitudes. In general, OA and SA are moderately positively correlated. We hypothesized that for people who score high in mindfulness, especially on measures of acceptance, the relationship between OA and SA would be weaker. In this study, we measured participants’ trait mindfulness and their OA and SA toward multiple attitude objects, and examined the moderation effect of mindfulness on the OA–SA relationship.

### Methods

#### Participants

One hundred and thirteen undergraduates (*M*_age_ = 19.10 years, *SD* = 1.48; 39 females; 79 White, 11 Black, 13 Asian, 6 Hispanic or Latino, 1 Asian Indian, 1 Pacific Islander, 2 Middle Eastern, 1 Caribbean, multiple selections were possible) from the University at Buffalo received course credit for participating. Participants completed the study online. We collected as many participants as we could until the last day of the semester in which the study was conducted.

#### Procedure

Participants first reported their trait mindfulness, and then reported their attitudes toward a series of different objects. To capture the awareness and the acceptance components of mindfulness we used the Philadelphia Mindfulness Scale (Cardaciotto et al., 2008). In addition, we used the Cognitive Fusion Questionnaire (Gillanders et al., 2014) and decentering subscale of Experience Questionnaire (Fresco et al., 2007), both of which should capture aspects of acceptance.^1^

### Materials

#### Philadelphia Mindfulness Scale

The Philadelphia Mindfulness Scale (PMS; Cardaciotto et al., 2008) is a 20-item measure of mindfulness that assesses two components of mindfulness, present moment awareness (e.g., “I am aware of what thoughts are passing through my mind.”; α = 0.803, *M* = 3.541, *SD* = 0.539) and acceptance [e.g., “I tell myself that I shouldn’t have certain thoughts.” (reversed); α = 0.806, *M* = 2.789, *SD* = 0.586]. Participants rate each item on a 5-point scale (1 = *never*; 5 = *very often*).

#### Cognitive Fusion Questionnaire

The Cognitive Fusion Questionnaire (CFQ; Gillanders et al., 2014) is a 7-item self-report measure of the tendency to “fuse” with or struggle with one’s thoughts (e.g., “I get so caught up in my thoughts that I am unable to do the things that I most want to do.”), and is one measure of the mindfulness-related concept of decentering/defusion (see Bernstein et al., 2015). The CFQ is strongly related to commonly-used measures of mindfulness, particularly the more “active” components that share conceptual parallels to acceptance (e.g., non-judging and non-reactivity; Naragon-Gainey and DeMarree, 2017b). Participants rate each item on a 7-point scale (1 = *never true*; 7 = *very true*). Because higher scores would typically be a negative indicator of mindfulness, we reverse coded CFQ items, to be consistent with the other measures. Consequently, higher scores on the CFQ in this study indicate higher mindfulness (α = 0.933, *M* = 4.236, *SD* = 1.420).

#### Experiences Questionnaire-Decentering

The Experiences Questionnaire (EQ; Fresco et al., 2007) is a measure of decentering and rumination. Participants in this sample completed the 11-item decentering subscale, which measures the ability to observe one’s thoughts and feelings as temporary and from objective perspective (e.g., “I can separate myself from my thoughts and feelings.”), and is another measure of the mindfulness-related concept of decentering/defusion (see Bernstein et al., 2015). The EQ is moderately to strongly related to commonly-used measures of mindfulness, particularly the more “active” components that share conceptual parallels to acceptance (e.g., non-judging and non-reactivity; Naragon-Gainey and DeMarree, 2017b). Participants rate each item on a 5-point scale (1 = *never*; 5 = *all the time*; α = 0.870, *M* = 3.215, *SD* = 0.649).

#### Attitude Objects

In order to maximize generalizability across the range of topics about which people have attitudes and to increase power by having within-subject replications, we generated a pool of topics to which participants could respond. There were 18 objects in the pool representing a range of topics: exercising, eating broccoli, drinking alcohol, using condoms, recycling, genetically modified foods, nuclear power, abortion, death penalty, gay marriage, African Americans, Hillary Clinton, Donald Trump, Walmart, Microsoft, library, exams, and conformity. Each participant was randomly presented with only 10 objects randomly selected from the attitude object pool in random order. Participants answered a series of questions regarding each attitude object before moving on to the next attitude object. Specifically, for each object, participants indicated their objective and subjective ambivalence using the scales described below.

#### Objective Ambivalence

To measure attitudinal ambivalence, for each object participants responded to six unipolar scales assessing positivity and negativity. Participants were asked “to what extent does each of the words below describe your attitude toward [topic]?”. Participants were presented with three positive (good, favorable, positive; α = 0.950, *M* = 1.678; *SD* = 1.028) and three negative (bad, unfavorable, negative; α = 0.927, *M* = 0.894; *SD* = 0.921) adjectives, in random order, and asked to rate each item on a 4-point scale (0 = *not at all*; 1 = *slightly*; 2 = *quite*; 3 = *extremely*). OA is computed from the separate positive and negative reactions using the commonly used Griffin formula: (positive reaction + negative reaction)/2 -| positive reaction - negative reaction| (Thompson et al., 1995). This formula considers both the intensity of positive and negative reactions (the average of both reactions) and the similarity of both reactions (by subtracting the absolute value of the discrepancy between positive and negative reactions) into account. This formula produces the highest OA score when the intensity of positive and negative reactions toward an object are relatively high and similar in magnitude, and produces the lowest ambivalence score when people’s evaluations are entirely one-sided (positive or negative). OA (*M* = -0.281; *SD* = 1.058) is normally distributed in this study and also in the studies presented later, because the skewness and kurtosis of OA for all studies fall within the acceptable range of +2 and -2 (George and Mallery, 2012).

#### Subjective Ambivalence

Participants then responded to four items assessing SA. The items were “when you think about [topic], you find yourself feeling: indecisive/confused/conflicted/torn between the two sides of the issue.” Participants rated each item on a 9-point scale (1 = *not at all*; 9 = *extremely;* α = 0.960; *M* = 2.997; *SD* = 2.161).

### Results

The data in this study were uniquely structured and traditional regression models would not be appropriate. First, participants responded to 10 different attitude objects each, and consequently those responses were not independent of each other. That is, there are meaningful individual differences that need to be modeled (e.g., Thompson and Zanna, 1995) to account for the non-independence of those observations and to facilitate generalization to the population from which participants were drawn. Second, just as participants are assumed to be sampled from a larger population, so too are the specific attitude objects to which participants responded sampled from a larger population, and each attitude object can potentially vary in meaningful ways (e.g., attitudes might systematically vary in their valence or ambivalence across attitude objects). By using a cross-classified model, in which each observation is “nested” within the participant providing that observation *and* within the specific attitude object that was being responded to, both of these sources of variability are explicitly modeled (see e.g., Judd et al., 2012).

To examine the main effect of mindfulness on OA and SA, we used the specified mindfulness measures or composites to predict OA and SA. To examine the moderation effect of mindfulness on SA, we used OA, the specified mindfulness measures or composites, and the interaction of OA and mindfulness to predict SA. In all models, both attitude object and subject were treated as random effects, so the intercepts can vary across different attitude objects and individuals. The results showed that in all models the intercepts significantly varied across attitude objects, all *Wald Z*s > 2.47, all *p*s < 0.002, and across participants, all *Wald Z*s > 5.53, all *p*s < 0.001. In addition, we also allowed the level 1 slope of OA predicting SA to vary across participants, as it is variability in this slope that we are interested in predicting from the mindfulness variables that are measured at level 2. However, we did not allow the level 1 slope of OA predicting SA to vary across attitude objects, because doing so produced errors in model convergence, potentially due to the relatively few attitude objects used.

We examined whether awareness and acceptance components of mindfulness predict OA and SA, and whether they moderate the OA–SA relationship. We combined three acceptance-related subscales (PMS-acceptance, CFQ, EQ) by averaging standardized scale scores to create an acceptance composite (α = 0.893), and PMS-awareness subscale as an awareness composite. The results of the main effect of individual full scales or subscales of mindfulness on OA and SA, and their moderation effect on the OA–SA relationship are available in Supplementary Materials.

#### Main Effects

We first tried to replicate the past research showing that mindfulness negatively predicts OA and SA (Haddock et al., 2017). To do this, we used cross-classified multilevel models as described above. First, we examined the main effect of awareness and acceptance components of mindfulness on OA and SA. In this model, both awareness and acceptance significantly negatively predicted OA (see Table 1). We then conducted parallel analyses predicting SA instead. In the model, both awareness and acceptance negatively predicted SA, but only acceptance significantly did so (see Table 1).

**Table 1:** Results of multilevel regression analyses examining the main effects of mindfulness on OA and SA.

	Parameter	*b*	*SE*	*t*	*p*
Study 1 (*N* = 113)	Awareness	–0.107	0.051	–2.098	0.038
OA	Acceptance	–0.173	0.070	–2.456	0.016
Study 1	Awareness	–0.186	0.118	–1.578	0.117
SA	Acceptance	–0.392	0.163	–2.406	0.018
Study 2 (*N* = 209)	Awareness	–0.060	0.047	–1.257	0.210
OA	Acceptance	–0.048	0.065	–0.733	0.465
Study 2	Awareness	–0.017	0.076	–0.222	0.825
SA	Acceptance	–0.223	0.105	–2.127	0.035

*OA, objective ambivalence; SA, subjective ambivalence*.

#### Moderation Effect

**FIGURE 1:**
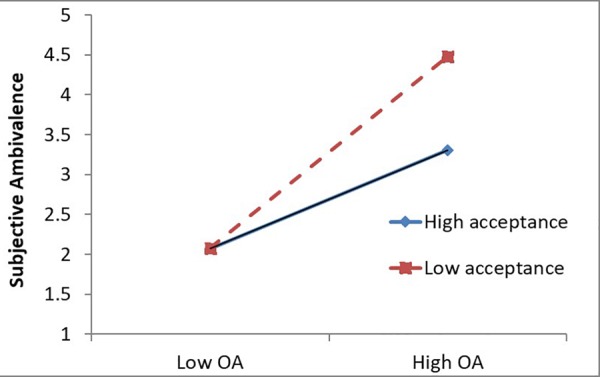
Interaction of objective ambivalence and acceptance composite predicting subjective ambivalence (Study 1). Graph plotted at ±1*SD* from the sample mean. OA, objective ambivalence.

The main purpose of this study is to examine whether mindfulness moderates the relationship between OA and SA. First, without controlling for other variables, OA positively predicted SA [*b* = 0.944, *SE* = 0.073, *t*(130.90) = 12.993, *p* < 0.001]. As predicted, when both mindfulness composites and their interactions with OA were added to the model, acceptance attenuated the relationship between OA and SA, but awareness did not (see Table 2). That is, for people with high acceptance, the relationship between OA and SA was weaker (see Figure 1).

**Table 2:** Results of multilevel regression analyses examining the interaction effects of mindfulness and OA/ambivalence manipulation in predicting SA/negative affect and associated simple slopes of OA/ambivalence manipulation on SA/negative affect at high and low levels of each mindfulness variable.

		Interaction effect				OA at low mind		OA at high mind	
	Parameter	*b*	*SE*	*t*	*p*	*b*	*SE*	*b*	*SE*
Study 1 *N* = 113 DV: SA	OA	0.925	0.070	13.161	0.000				
	Awareness	–0.108	0.100	–1.084	0.281				
	Acceptance	–0.272	0.138	–1.971	0.051				
	Awareness × OA	0.072	0.068	1.064	0.290	0.853***	0.099	0.998***	0.097
	Acceptance × OA	–0.274	0.094	–2.900	0.004	1.199***	0.116	0.651***	0.120
Study 2 *N* = 209 DV: SA	OA	0.774	0.049	15.951	0.000				
	Awareness	0.014	0.061	0.222	0.824				
	Acceptance	–0.172	0.083	–2.075	0.039				
	Awareness × OA	0.101	0.050	1.996	0.047	0.673***	0.071	0.874***	0.069
	Acceptance × OA	–0.012	0.069	–0.169	0.866	0.785***	0.083	0.762***	0.086
Study 3 *N* = 278 DV: SA	Condition	0.925	0.174	5.324	0.000				
	Awareness	–0.250	0.136	–1.829	0.068				
	Acceptance	–0.082	0.143	–0.570	0.569				
	Awareness × Condition	0.177	0.204	0.869	0.386	0.774**	0.245	1.075***	0.246
	Acceptance × Condition	–0.649	0.240	–2.710	0.007	1.412**	0.254	0.438	0.246
Study 4 *N* = 354 DV: SA	Condition	0.098	0.152	0.644	0.520				
	Awareness	0.191	0.118	1.615	0.107				
	Acceptance	–0.171	0.154	–1.109	0.268				
	Awareness × Condition	0.108	0.178	0.604	0.546	0.005	0.215	0.190	0.216
	Acceptance × Condition	0.379	0.242	1.570	0.117	–0.145	0.215	0.340	0.218
Study 4 DV: Negative affect	Condition	0.392	0.147	2.663	0.008				
	Awareness	0.057	0.115	0.494	0.622				
	Acceptance	–0.481	0.150	–3.214	0.001				
	Awareness × Condition	–0.184	0.173	–1.060	0.290	0.579**	0.208	0.248	0.209
	Acceptance × Condition	0.523	0.234	2.230	0.026	0.077	0.208	0.750***	0.210
Study 5 *N* = 372 DV: SA	Condition	0.939	0.166	5.664	0.000				
	Awareness	–0.155	0.140	–1.109	0.268				
	Acceptance	–0.131	0.161	–0.814	0.416				
	Awareness × Condition	–0.081	0.190	–0.429	0.669	1.010***	0.235	0.867***	0.262
	Acceptance × Condition	0.078	0.230	0.338	0.735	0.881***	0.235	0.996**	0.234
Study 5 *N* = 372 DV: Negative affect	Condition	0.679	0.126	5.373	0.000				
	Awareness	–0.253	0.107	–2.369	0.018				
	Acceptance	–0.086	0.123	–0.700	0.484				
	Awareness × Condition	0.260	0.145	1.794	0.074	0.451*	0.180	0.908***	0.180
	Acceptance × Condition	–0.374	0.175	–2.135	0.033	0.970***	0.180	0.396*	0.179

*OA, objective ambivalence; SA, subjective ambivalence. All predictors were standardized. ^∗^p < 0.05, ^∗∗^p < 0.01, ^∗∗∗^p < 0.001*.

#### Exploratory Analyses

It is possible that the moderating effects of acceptance should only occur if someone was aware of their internal states, because awareness might be the precursor of acceptance. Thus, we examined the 3-way interaction between OA, awareness, and acceptance on SA. Failing to support this prediction, there were no significant 3-way interactions in this study and the following studies, all *p*s > 0.14.

### Discussion

In Study 1, we replicated past work from Haddock et al. (2017) showing that mindfulness, both the acceptance and awareness components, negatively predicted OA and SA. Consistent with our primary hypothesis, the results showed that acceptance moderated the relationship between OA and SA. As people’s endorsement of acceptance-related concepts increased, the relationship between OA and SA became weaker. This may suggest that people who are more open and accepting of their thoughts and feelings are more tolerant of the mixed reactions they have, and therefore report less psychological discomfort. So, it seems that mindfulness, especially acceptance, may buffer the effect of the objective ambivalence on subjective ambivalence. However, this is a single study, so it is important to replicate this effect to determine the extent to which it is robust.

Although the conceptualization of mindfulness in terms of awareness and acceptance has precedent in the literature, and makes conceptual sense based on a great deal of work (e.g., Bishop et al., 2004; Coffey et al., 2010), this is not the only perspective. At the request of a reviewer, we examined whether the data from this study and the following studies supported this two-factor distinction. Specifically, we ran an exploratory factor analysis to see whether a two-factor structure mapped onto our awareness and acceptance composites. The two-factor solutions we found across all five studies seemed to represent the direction of item coding (i.e., forward versus reverse coded items), which shares only some conceptual overlap with awareness versus acceptance (e.g., all items of PMS-acceptance, CFQ, and FFMQ-non-judging are reverse coded, making it difficult to interpret the reason for their loadings on the same factor). Many of the existing mindfulness measures have been criticized on their measurement properties (Van Dam et al., 2010, 2012, 2017; Grossman, 2011), including issues with the direction of item coding (e.g., Van Dam et al., 2012; Aguado et al., 2015). Interested readers are directed to the Supplementary Materials if they would like to examine full analyses with each of the individual subscales in any of our studies.

## Study 2

The goal of this study is to try to replicate the results we found in Study 1 with a larger sample size. The procedure, attitude objects and attitude measures are the same as Study 1, except as noted below. In this Study, we dropped CFQ and EQ measures used in Study 1. Though EQ and CFQ can closely capture the concept of acceptance, they are more commonly used in the clinical psychology literature, and are not used extensively in the non-clinical literature on mindfulness. Instead, in this study, we included the more commonly used Five Facet Mindfulness Questionnaire, which was also one of the mindfulness measure used in Haddock et al. (2017), to more fully capture the breadth of mindfulness, although we still focus on awareness and acceptance.

### Methods

#### Participants

Two hundred and nine undergraduates (*M*_age_ = 18.79 years, *SD* = 1.03; 117 females; 118 White, 14 Black, 1 American Indian, 64 Asian, 15 Hispanic or Latino, 8 Asian Indian, 2 Other Pacific Islander, multiple selections were possible) from the University at Buffalo received research credit for participating. Participants completed the study online. Although we did not conduct a formal power analysis, our plan was to roughly double the sample size of Study 1.

#### Procedure

The procedure of this study is the same as Study 1. Participants reported trait mindfulness first, then reported their attitudes toward 10 out of the same 18 attitude objects used in Study 1.^2^

### Materials

#### Philadelphia Mindfulness Scale

The awareness (α = 0.869, *M* = 3.441, *SD* = 0.643) and acceptance (α = 0.846, *M* = 2.725, *SD* = 0.650) subscales of the Philadelphia Mindfulness Scale were again used in this study.

#### Five Facet Mindfulness Questionnaire

The Five Facet Mindfulness Questionnaire (FFMQ; Baer et al., 2006) is a 39-item measure of mindfulness with 5 subscales: observe, describe, acting with awareness, non-judging, and non-reactivity. The observe facet (α = 0.852, *M* = 3.164, *SD* = 0.695) measures the tendency to attend to one’s current sensations, feelings, and thoughts. The describe facet (α = 0.810, *M* = 3.071, *SD* = 0.638) measures the ability to label or describe one’s feelings and thoughts with words. The acting with awareness (α = 0.881, *M* = 3.120, *SD* = 0.715) facet measures the ability to be aware of one’s behavior and not just automatically do things without awareness. The non-judging facet (α = 0.911, *M* = 3.169, *SD* = 0.811) measures the ability to be open to one’s experience without criticism. The non-reactivity facet (α = 0.780, *M* = 2.986, *SD* = 0.605) measures the ability to disengage and not let oneself be affected by one’s own feelings and thoughts. A sample item is “I perceive my feelings and emotions without having to react to them.” (non-reactivity). Participants rate each item on a 5-point scale (1 = *never*; 5 = *always*). Based on a conceptual understanding of these subscales, we considered the observing subscale to represent people’s awareness. Consistent with this, in past research items from the observing facet have explicitly been treated as present moment awareness (Cameron and Fredrickson, 2015). In contrast, because the non-judging and non-reactivity subscales represent people’s openness toward one’s experience and the ability to disengage and not be affected by their thoughts and feelings, we viewed these subscales are relevant to acceptance. Indeed, items from the non-judging facet have explicitly been treated as acceptance (Cameron and Fredrickson, 2015). In Haddock et al. (2017), they also used FFMQ to measure mindfulness, and the results showed that only acting with awareness negatively correlated with OA, while describe and acting with awareness both negatively predicted SA.

#### Objective Ambivalence

For each issue, participants indicated their positive reaction (α = 0.954, *M* = 2.609, *SD* = 0.980) and negative reaction (α = 0.944, *M* = 1.954, *SD* = 0.910) using the same measure as Study 1 to compute OA (*M* = 0.860, *SD* = 1.098), but participants rated each item on a 4-point scale (1 = *not at all*; 4 = *extremely*).

#### Subjective Ambivalence

Participants completed the same measure of SA as Study 1, except that we dropped the “confused” item, because we wanted to make room for other measures we added. The three items we kept are more directly asked about conflicted feelings. Participants rated each item on a 7-point scale (1 = *not at all*; 7 = *extremely*; α = 0.955, *M* = 2.865, *SD* = 1.785).

### Results

The data structure and design of this study are the same as Study 1, so our analysis strategy is the same as well. In all models the intercepts significantly varied across attitude objects, all *Wald Z*s > 2.43, all *p*s < 0.015, and across participants, all *Wald Z*s > 6.80, all *p*s < 0.001. We still treated level 1 slope of OA predicting SA as random and varied across participants. Again, we did not allow the level 1 slope of OA predicting SA to vary across attitude objects because doing so produced model convergence errors. We first examined the main effect of awareness and acceptance component of mindfulness on OA and SA, then examined the moderation effect. We combined three acceptance-related subscales (PMS-acceptance, FFMQ-non-judging, and FFMQ-non-reactivity) by averaging standardized scale scores to create an acceptance composite (α = 0.865), and combined two awareness-related subscales (PMS-awareness and FFMQ-observe) subscale as awareness composite (α = 0.913). The results of the main effect of individual full scales or subscales of mindfulness on OA and SA, and their moderation effect on the OA–SA relationship are available in Supplementary Materials.

#### Main Effects

We examined the main effect of the awareness and acceptance composites on OA and SA. Neither awareness nor acceptance predicted OA (see Table 1). When predicting SA, acceptance significantly negatively predicted SA, but awareness did not (see Table 1). When we examined the individual subscales of the FFMQ as in Haddock et al. (2017), we found when all individual subscales of the FFMQ were included in the model to predict SA, none of the subscales significantly predict SA (see Supplementary Materials for full analyses with subscales).

#### Moderation Effect

**FIGURE 2:**
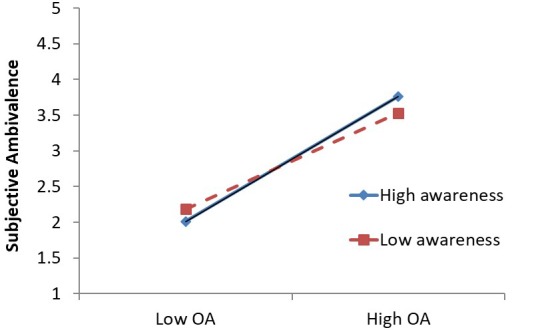
Interaction of objective ambivalence and awareness composite predicting subjective ambivalence (Study 2). Graph plotted at ±1*SD* from the sample mean. OA, objective ambivalence.

First, without other variables in the model, OA positively predicted SA [*b* = 0.778, *SE* = 0.049, *t*(216.56) = 15.936, *p* < 0.001]. However, in this study, acceptance did not attenuate the relationship between OA and SA, though awareness did moderate the relationship between OA and SA (see Table 2). Specifically, for people with high awareness, the relationship between OA and SA was stronger (see Figure 2), suggesting that awareness was *strengthening* the OA–SA relationship.

### Discussion

In this study, we partially replicated the main effect of mindfulness on OA and SA. We found that only acceptance negatively predicted SA. However, we did not replicate the key moderation pattern we observed in Study 1. That is, acceptance did not predict an attenuated relationship between OA and SA in this study. It is unclear why this effect did not replicate across studies, particularly given how similar the methods were. We will reserve an extended discussion of the consistency or inconsistency of this and any other effects until the general discussion. In addition, in this study we found that awareness predicted a stronger relationship between OA and SA. Although this is speculative, this finding may suggest that people with high awareness are more attentive to the mixed reaction they have, and therefore these mixed reactions are more likely to lead to stronger psychological discomfort. Although this effect of awareness was not significant in Study 1, the pattern of results was consistent with the pattern observed in this study.

The first two studies both used entirely correlational methods, and consequently are not without their limitations. Most notably, by using familiar attitude objects, it is unclear whether we were capturing psychological processes that were happening in the moment or those that had occurred previously. With an existing attitude, people may have had many previous experiences in which the ambivalent attitude was activated or in awareness, and people may respond to that ambivalence in a variety of ways, such as through decision delay, careful elaboration, reappraisal processes, and so forth. These previous experiences may depend on an individual’s level of mindfulness and could affect both mean levels of OA and SA as well as the OA–SA relationship. For example, one possibility is that people with high mindfulness may be more aware of the mixed reactions they had, which may have elicited ambivalence reducing behavior before they participated in these studies. Therefore, the mean levels of OA and SA may be lower for people with high mindfulness (as in Haddock et al., 2017). Also, if the specific responses to ambivalence differed systematically as a function of a person’s level of mindfulness (e.g., if people low in mindfulness responded with delay and distraction, but people high in mindfulness responded with careful elaboration and reappraisal processes), then the properties of the attitudes of people high and low in mindfulness would likely diverge over time. Therefore, it is possible that in a correlational study, any differences observed (such as those in Study 1), would be due to these past psychological processes, not those taking place in the moment as we initially hypothesized. To address this concern, we manipulated ambivalence in Study 3 and used a novel topic so that whatever psychological processes are happening are limited to those that occur in the course of the study. Thus, we sought to replicate Study 1 by showing that acceptance attenuates the OA–SA relationship even when OA is experimentally induced, and also to see whether the results that awareness strengthens the OA–SA relationship in Study 2 can be replicated.

## Study 3

In this study, we manipulated ambivalence by having participants read either relatively ambivalent or univalent information about a consumer product. Although the acceptance component of mindfulness attenuated the relationship between OA and SA in Study 1, it did not do so in Study 2. It is not clear if this reduced relationship, if genuine, reflected processes that had happened in the moment or that had occurred previous to the study, particularly because each of the topics were ones that participants had likely considered previously, and as described above mindfulness may affect both mean levels of OA and SA as well as the OA–SA relationship in pre-existing attitudes. In Study 3, rather than relying on pre-existing attitudes, we introduced a new topic and manipulated the ambivalence of information provided about a new product. By introducing a new topic, we can remove any ambivalence-reduction efforts that might have occurred previously, and because the study does not provide any obvious ambivalence-reduction opportunities, we could more precisely examine whether mindfulness would moderate the extent to which people *feel* ambivalent given the same objective level of ambivalence. We hypothesized that even when exposed to highly ambivalent information, people with high acceptance would not experience *subjective ambivalence* to the same extent as low acceptance people feel.

### Methods

#### Participants

Two hundred and seventy-eight undergraduates (*M*_age_ = 18.97 years, *SD* = 1.39; 146 females; 162 White, 37 Black, 64 Asian, 21 Hispanic or Latino, 13 Asian Indian, 5 unreported, multiple selections were possible) from University at Buffalo received research credit for participating. Participants completed the study in the lab. This is the first use of the paradigm, so we did not have a clear rationale to determine the sample size. We planned to collect data for 2 weeks or until we reached at least 200 participants.

#### Procedure

Participants first reported their trait mindfulness using the Philadelphia Mindfulness Scale (Cardaciotto et al., 2008), Five Facet Mindfulness Questionnaire (Baer et al., 2006), and an in-development measure of meta-awareness (Naragon-Gainey and DeMarree, unpublished). Then, they were randomly assigned to one of two conditions in which they read information about Moro Bars, which were described as a snack food from New Zealand that would be test-marketed in several United States Markets. In the ambivalent condition, participants read strong pro-Moro bar information and strong anti-Moro bar information. In the univalent control condition, participants read the same strong pro-Moro bar information but also read weak anti-Moro bar information. After the manipulation, they reported their attitudes about Moro bars.^3^

### Materials

#### Philadelphia Mindfulness Scale

The awareness (α = 0.812, *M* = 3.716, *SD* = 0.544) and acceptance (α = 0.873, *M* = 2.664, *SD* = 0.691) subscales of the Philadelphia Mindfulness Scale were again used in this study.

#### Five Facet Mindfulness Questionnaire

The observe (α = 0.811, *M* = 3.389, *SD* = 0.662), describe (α = 0.866, *M* = 3.291, *SD* = 0.698), acting with awareness (α = 0.867, *M* = 3.090, *SD* = 0.703), non-judging (α = 0.895, *M* = 3.185, *SD* = 0.785), non-reactivity (α = 0.751, *M* = 3.084, *SD* = 0.540) subscales of the Five Facet Mindfulness Questionnaire were again used in this study.

#### Meta-Awareness

We used the meta-awareness subscale (α = 0.913, *M* = 5.511, *SD* = 0.876) of an in-development measure of decentering (Naragon-Gainey and DeMarree, 2019). This subscale is an 8-item measure of people’s present-moment awareness of their mental states (i.e., their inner experiences). A sample item is “I notice how my thoughts and feelings come and go.” Participants rated each item on a 7-point scale (1 = *strongly disagree*; 7 = *strongly agree*). Most other measures of awareness, including both the PMS-Awareness and FFMQ-Observing, measure awareness toward inner experiences, physical experiences, and experience of the external world. Because of this, if they fail to moderate OA–SA relationships, it would be unclear if this were because awareness of one’s inner states does not moderate this effect or because these measures of awareness were too broad to specifically capture the relevant type of awareness (i.e., inner awareness).

#### Ambivalence Manipulation

The messages we used were adapted from Belding et al. (unpublished). The topic was Moro Bars, a snack bar from New Zealand that people in the United States would be unfamiliar with. In order to make participants take the study seriously, they were told that this snack might be introduced to the United States in the future, and that the product was going to be test marketed in several United States cities, including their city. Participants were randomly assigned to either the ambivalent or control condition. In both conditions, participants were presented with strong arguments in favor of Moro Bars which included five arguments in support of Moro Bars (e.g., “Moro Bars contain tryptophan, which can result in decreased anxiety.”) and five arguments against Moro Bars, which were manipulated to be either compelling (to create a relatively ambivalent attitude) or not compelling (to create a relatively univalent attitude). In the ambivalent condition, participants read strong arguments against Moro Bars which included five reasons why Moro Bars are bad (e.g., “Moro Bars contain high amounts of saturated and trans fats.”). In the relatively low ambivalence condition, participants read weak arguments against Moro Bars (e.g., “Moro Bars have the same color and appearance as poo.”). The order of presentation of the two sets of arguments was randomized. The logic of this induction is that the conditions were reasonably equivalent in their length and two-sided nature, but if participants read the information carefully, then their resultant attitude should be consistent with the intended conditions.

#### Objective Ambivalence

For each issue, participants indicated their positive reaction (α = 0.940, *M* = 4.481, *SD* = 1.360) and negative reaction (α = 0.931, *M* = 3.245, *SD* = 1.497) using the same measure as Study 2 to compute OA (*M* = 1.564, *SD* = 1.816), but participants rated each item on a 7-point scale (1 = *not at all*; 7 = *very much*).

#### Subjective Ambivalence

The SA was measured using the same items as Study 2 (α = 0.913, *M* = 2.950, *SD* = 1.528).

### Results

#### Manipulation Check

We first examined whether the ambivalence manipulation was successful using an independent samples *t*-test. As expected, people in ambivalent condition (*M* = 2.077, *SD* = 1.674, *SE* = 0.152) reported higher OA than people in control condition (*M* = 1.169, *SD* = 1.826, *SE* = 0.146), *t*(276) = 4.261, *p* < 0.001. We also tested whether the ambivalence manipulation was equally effective across levels of mindfulness using a series of Condition × Mindfulness regression analyses. That is, we used condition, the mindfulness variables specified, and the interaction (cross-product) of the condition and mindfulness measures to predict OA. The results showed that mindfulness using full scales, subscales, or composites did not moderate the effect of the manipulation on OA, |*t*s|≤ 1.60, *p*s ≥ 0.111. That is, across levels of mindfulness, the ambivalence manipulation was equally effective.

#### Moderation Effect

**FIGURE 3:**
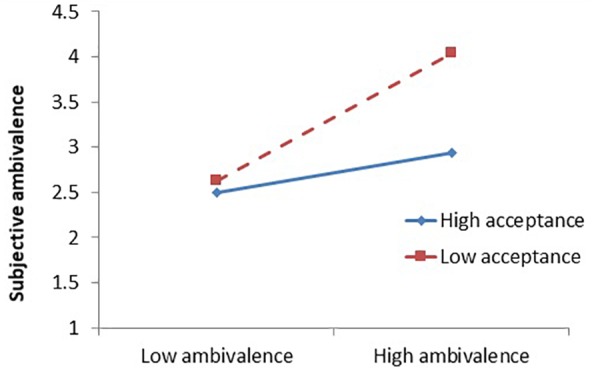
Interaction of ambivalence manipulation and acceptance composite predicting subjective ambivalence (Study 3). Graph plotted at ±1*SD* from the sample mean.

Next, we examined whether, consistent with our hypothesis and the results of Study 1, the acceptance component of mindfulness attenuated the effect of ambivalence manipulation on SA. First, we combined three acceptance-related subscales (PMS-acceptance, FFMQ-non-judging, and FFMQ-non-reactivity) by averaging standardized scale scores to create an acceptance composite (α = 0.902), and also combined three awareness-related subscales (meta-awareness, PMS-awareness, and FFMQ-observe) to create an awareness composite (α = 0.921). Analyses were conducted in regression with the condition, the mindfulness variables specified, and the interaction (cross-product) of the condition and mindfulness measures predicting SA. Note that the meta-awareness measure did not moderate the OA–SA relationship when analyzed as an isolated subscale (*b* = -0.085, *SE* = 0.178, *p* = 0.632). When both composites were in the model, interacting with the condition to predict SA, acceptance composite significantly moderated the effect of the ambivalence manipulation on SA (see Table 2). As people’s acceptance of their feelings and thoughts increased, people were less likely to experience an increase in conflicted feelings about Moro bars after reading ambivalent instead of a univalent message (see Figure 3). The results of the moderation effect of individual full scales or subscales of mindfulness are available in Supplementary Materials.

### Discussion

In this study, using manipulated rather than measured OA, we replicated the effects observed in Study 1. Specifically, we found that acceptance aspects of mindfulness predicted attenuated effects of an ambivalence manipulation on SA. That is, as acceptance increased, people’s feelings of ambivalence were less strongly related to the ambivalence of information. These effects do not appear to be due to differences in people’s understanding of the information provided, as the ambivalence manipulation induced the same level of OA for people with high and low mindfulness. Therefore, mindfulness buffered the effect of ambivalence manipulation on the experience of conflict, but not on the presence of conflicting reactions.

## Study 4

In Study 3, the experimental manipulation of ambivalence was related to feelings of ambivalence for people low, but not high in acceptance. Indeed, among people high in acceptance, the ambivalence manipulation had no effect on subjective ambivalence. However, one possibility is that the topic was insufficiently important, and that the effects of objective ambivalence on a more meaningful topic would overpower the potential effects of acceptance. In this study, we sought to conceptually replicate the effect observed in Study 3 using a more societally important topic to manipulate ambivalence, GMO foods. Although the topic is likely somewhat familiar to participants, we hoped the topic would be novel enough for them to respond to arguments to produce the intended pattern of ambivalence. We also added another dependent variable, negative affect, to directly measure psychological discomfort.

We initially ran a pilot study for the materials used in this study and failed to find a strong effect of the ambivalence manipulation. To increase our chances of creating a meaningful study, we edited the original arguments to make them more clear and easier to understand and added a measure of people’s likelihood of thinking carefully – the need for cognition scale (Cacioppo et al., 1984). Argument quality differences, such as those that form the basis of the current manipulation, are more likely to emerge when people are thinking carefully (Petty and Cacioppo, 1986), including as assessed by the need for cognition scale (e.g., Luttrell et al., 2017). Thus, including the measure provided a potential way to predict the people for whom the ambivalence manipulation was likely to be successful.

### Methods

#### Participants

Three hundred and fifty-four undergraduates (*M*_age_ = 19.15 years, *SD* = 2.45; 159 females; 173 White, 39 Black, 1 American Indian, 119 Asian, 29 Hispanic or Latino, 23 Asian Indian, 5 unreported, multiple selections were possible) from University at Buffalo received research credit for participating. Participants completed the study online. The sample size was determined by the maximum number of participants we could run in 2 weeks. The resultant sample size had a power of 0.87 to detect an interaction effect of the size observed in Study 3 based on a Monte Carlo simulation study conducted in Mplus using the parameters observed in Study 3.

#### Procedure

Participants first reported their trait mindfulness. Then, they were randomly assigned to one of two conditions in which they read low ambivalent or high ambivalent information about genetically modified foods (GM foods). Then, they reported their attitudes toward GM foods, negative affect, and need for cognition.^4^

### Materials

#### Philadelphia Mindfulness Scale

The awareness (α = 0.849, *M* = 3.560, *SD* = 0.604) and acceptance (α = 0.872, *M* = 2.694, *SD* = 0.675) subscales of the Philadelphia Mindfulness Scale were again used in this study.

#### Five Facet Mindfulness Questionnaire

The observe (α = 0.821, *M* = 3.343, *SD* = 0.656), describe (α = 0.766, *M* = 3.132, *SD* = 0.579), acting with awareness (α = 0.832, *M* = 2.904, *SD* = 0.621), non-judging (α = 0.896, *M* = 2.957, *SD* = 0.764), non-reactivity (α = 0.767, *M* = 3.146, *SD* = 0.566) subscales of the Five Facet Mindfulness Questionnaire were again used in this study.

#### Meta-Awareness

The meta-awareness subscale (α = 0.932, *M* = 5.227, *SD* = 1.033) of an in-development measure of decentering was again used in this study.

#### Ambivalence Manipulation

The messages we used were adapted from Luttrell (unpublished). Participants were randomly assigned to either the ambivalent or control condition. Parallel to the manipulation used in Study 3, in the ambivalent condition participants read an article that presented 5 strong arguments against GM foods (e.g., These crops can cause harm to animals. Though pest-resistant crops target specific pests that destroy crops, scientists can’t be sure that only the crop-damaging organisms are affected.) and 5 strong arguments in favor of GM foods (e.g., Global nutrition can benefit from genetically modified foods. For example, to help address a vitamin A deficiency for children in Southeast Asia, GM rice plants were developed that contain high amounts of vitamin A.). In the control condition, participants read an article that presented 5 weak arguments against GM foods (e.g., These crops can make farm landscapes less beautiful. Genetic modification makes plants all look very similar to each other, depriving these landscapes of natural variation. The view of farms will be too uniform, boring, and unnatural.) and the 5 strong arguments in favor of GM foods. Again, if participants read the information carefully, we expected that they would experience higher OA in the ambivalent condition than in the control condition.

#### Objective Ambivalence

Participants indicated their positive reaction (α = 0.920, *M* = 4.174, *SD* = 1.353) and negative reaction (α = 0.919, *M* = 3.694, *SD* = 1.386) using the same measure as Study 3 to compute OA (*M* = 2.057, *SD* = 1.732).

#### Subjective Ambivalence

Participants reported their SA using the measure as Study 3 (α = 0.885, *M* = 3.848, *SD* = 1.433).

#### Negative Affect

In this study, we added a measure of issue-related negative affect. Some conceptualizations of subjective ambivalence emphasize the affective nature of this experience (van Harreveld et al., 2009, 2015), so direct measures of this affect may supplement the findings from the SA measures described above. Participants responded to the question “To what extent do you experience each of the emotions below when you think about GM foods?” We selected 11 negative affect terms [tense, anxious, angry, fearful, uncomfortable, uneasy, bothered, agitated, regret, upset, and distress; items adapted from Nordgren et al. (2006) and Rydell et al. (2008); α = 0.960, *M* = 2.640, *SD* = 1.408], along with some filler positive affect terms (enthusiastic, inspired, excited, and interested). Participants rated each item on a 7-point scale (1 = *not at all*; 7 = *very much*).

#### Need for Cognition Scale

Participants completed the need for cognition scale (Cacioppo et al., 1984), an 18-item measure of individual differences in people’s enjoyment of effortful and complicated cognitive activities like thinking and problem solving. Each item was answered on a 7-point scale (1 = *strongly disagree*; 7 = *strongly agree*; α = 0.816, *M* = 4.189, *SD* = 0.627).

### Results

#### Manipulation Check

We first examined whether the ambivalence manipulation was successful by submitting OA scores to an independent samples *t*-test. As expected, people in ambivalent condition (*M* = 2.264, *SD* = 1.631, *SE* = 0.125) reported higher OA than people in control condition (*M* = 1.866, *SD* = 1.804, *SE* = 0.133), *t*(276) = 2.170, *p* = 0.030. We also tested whether the ambivalence manipulation was equally effective across levels of mindfulness. The results showed that mindfulness using full scales, subscales, or composites did not moderate the effect of the ambivalence manipulation on OA, |*t*s|≤ 1.565, *p*s ≥ 0.118. That is, the ambivalence manipulation appeared to be equally effective across participants’ level of mindfulness. However, need for cognition did predict the effectiveness of ambivalence manipulation, *B* = 0.491, *SE* = 0.185, *t*(350) = 2.656, *p* = 0.008. That is, for people with high need for cognition, the ambivalence manipulation impacted OA as intended [*B* = 0.854, *SE* = 0.263, *t*(350) = 3.248, *p* = 0.001], but for people with low need for cognition, the ambivalence manipulation did not impact OA [*B* = -0.127, *SE* = 0.257, *t*(350) = -0.494, *p* = 0.621].

#### Moderation Effect

Next, we examined whether the acceptance component of mindfulness attenuated the effect of the ambivalence manipulation on SA and negative affect. Like previous studies, we combined the three acceptance-related subscales to create an acceptance composite (α = 0.888), and also combined three awareness-related subscales to create an awareness composite (α = 0.935). When both composites were in the model predicting SA, the results showed that neither the acceptance nor the awareness composite moderated the effect of the ambivalence manipulation on SA. If anything, there was a non-significant trend opposite to predictions such that that acceptance actually enhanced the effect of the manipulation on SA (see Table 2).

**FIGURE 4:**
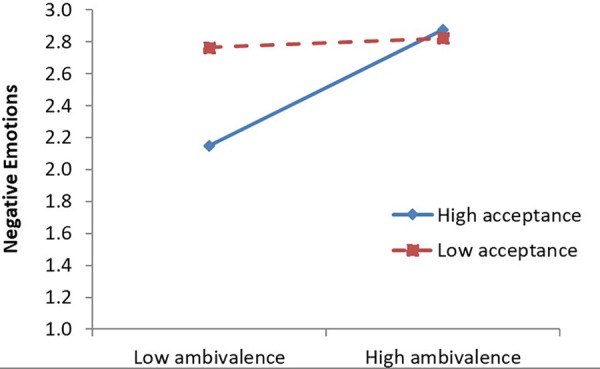
Interaction of ambivalence manipulation and acceptance composite predicting negative affect (Study 4). Graph plotted at ±1*SD* from the sample mean.

When a parallel analysis was conducted predicting negative affect, acceptance significantly strengthened the effect of the ambivalence manipulation on negative affect (see Table 2). That is, contrary to predictions, as people’s acceptance toward their feelings and thoughts increased, people’s experience of being torn and conflicted about GM foods was more responsive to the ambivalence versus univalence of the information in the message (see Figure 4). The results of the moderation effect of individual full scales or subscales of mindfulness are available in Supplementary Materials.

#### Exploratory Analyses

There may be some concerns that because GM foods is a controversial topic and pre-existing attitudes toward GM foods may affect the effectiveness of the manipulation. Although we did not measure pre-message attitudes in this study, we used a correlational approach to examine the interaction effect between measured OA (instead of the manipulation) and mindfulness on SA. There was no significant moderation effect of either the acceptance or awareness composite (*p*s > 0.23). However, a parallel analysis conducted predicting negative affect showed that the awareness composite predicted a smaller OA–SA relationship (*B* = -0.305, *SE* = 0.088, *t* = -3.453, *p* = 0.001), but the acceptance composite did not (*p* = 0.61). We also examined whether need for cognition would interact with awareness or acceptance and then moderate the effect of ambivalence manipulation on SA. However, the three-way interaction between manipulation, need for cognition, and the awareness or acceptance composite did not predict either SA (*p*s > 0.6) or negative affect (*p*s > 0.4).

### Discussion

In this study, we failed to replicate the results we found in the previous experimental study, and actually tended to find the opposite – that acceptance strengthened the effect of an ambivalence manipulation on SA and negative affect. Although, in this study, the ambivalence manipulation was not equivalently strong for all participants, as we found it was more effective for people high in need for cognition. When we added need for cognition to the primary analyses, the 3-way interaction between the ambivalence manipulation, need for cognition, and awareness or acceptance composite did not predict SA nor negative affect. In this study, the effect of ambivalence manipulation on OA was generally weaker than Study 3 (*d* = 0.51 in Study 3 vs. *d* = 0.23 in this study), which could be because the manipulation used was less effective or because it is more difficult to move people’s ambivalence on a relatively more familiar topic. Thus, it is unclear whether the failure to replicate the result of Study 3 was due to instability in the underlying effect (or non-effect), or a failure to create the conditions needed to find the effect. To more adequately address this ambiguity, we preregistered and conducted a final study, which was a very close replication of Study 3.

## Study 5

The goal of this study is to test whether we can replicate the results of Study 3 using an independent sample. The procedure, manipulation, and measures in this study are the same as Study 3 except as noted below. We also pre-registered this study on AsPredicted.org^5^.

### Methods

#### Participants

Three hundred and seventy-two undergraduates (*M*_age_ = 19.16 years, *SD* = 2.97; 227 females; 221 White, 41 Black, 96 Asian, 36 Hispanic or Latino, 2 Asian Indian, 1 Native Hawaiian, 2 unreported, multiple selections were possible) from University at Buffalo received research credit for participating. Participants completed the study online. The sample size was based on a Monte Carlo simulation study conducted in Mplus. Population parameters were estimated from the parameters obtained in Study 3, which used the same study materials as the current study. Based on this simulation, a sample size of 380 provided power = 0.90 to detect the predicted interaction effect at α = 0.05. Our preregistered data collection rule was to collect as many participants as possible, up to 500, before an internally set September 20th, 2018 deadline, with a minimum of 380 participants. Ultimately, we were 8 participants short of the minimum enrollment target on the target date, but we halted enrollment due to extremely slow signups (most interested participants appeared to have already completed the study).

#### Procedure

The procedure was the same as Study 3, but in this study, we also measured negative affect and need for cognition as we did in Study 4. First, participants reported their trait mindfulness, then they were randomly assigned to read either relatively ambivalent or unambivalent information about Moro Bars using the same materials employed in Study 3. After the ambivalence manipulation, participants reported their attitudes, negative affect, and need for cognition.

### Materials

#### Mindfulness

We used the same mindfulness measures as Study 3 and 4. Reliability for the measures was consistent with earlier studies (PMS-awareness: α = 0.842, *M* = 3.646, *SD* = 0.628; PMS-acceptance: α = 0.866, *M* = 2.596, *SD* = 0.698; FFMQ-observe: α = 0.802, *M* = 3.447, *SD* = 0.688; FFMQ-describe: α = 0.849, *M* = 3.124, *SD* = 0.720; FFMQ-acting with awareness: α = 0.860, *M* = 3.018, *SD* = 0.717; FFMQ-non-judging: α = 0.903, *M* = 3.000, *SD* = 0.847; FFMQ-non-reactivity: α = 0.734, *M* = 3.067, *SD* = 0.573; meta-awareness: α = 0.908, *M* = 5.382, *SD* = 0.948).

#### Ambivalence Manipulation

Same manipulation as Study 3.

#### Objective Ambivalence

Participants indicated their positive reaction (α = 0.935, *M* = 4.221, *SD* = 1.454) and negative reaction (α = 0.905, *M* = 3.580, *SD* = 1.528) using the same measure as Study 3 to compute OA (*M* = 1.695, *SD* = 1.866).

#### Subjective Ambivalence

Same measure as Study 3 (α = 0.918, *M* = 3.226, *SD* = 1.665).

#### Negative Affect

Same measure as Study 4 (α = 0.952, *M* = 2.108, *SD* = 1.285).

#### Need for Cognition Scale

Same measure as Study 4 (α = 0.860, *M* = 4.292, *SD* = 0.765).

### Results

#### Manipulation Check

We first examined whether the ambivalence manipulation was effective by submitting OA to an independent samples *t*-test. As expected, people in the ambivalent condition (*M* = 2.055, *SD* = 1.764, *SE* = 0.130) reported higher OA than people in the control condition (*M* = 1.342, *SD* = 1.901, *SE* = 0.139), *t*(370) = 3.748, *p* < 0.001. We also tested whether the ambivalence manipulation was equally effective across levels of mindfulness. The results showed that mindfulness did not moderate the effect of the manipulation on OA, |*t*s|≤ 0.430, *p*s ≥ 0.260. In addition, need for cognition did not moderate the effect of the manipulation on OA [*B* = -0.144, *SE* = 0.193, *t*(368) = -0.747, *p* = 0.455], either.

#### Moderation Effect

Next, we examined whether mindfulness moderated the effect of the ambivalence manipulation on SA. We made an awareness composite by combining 3 awareness related subscales (PMS-awareness, FFMQ-observe, and meta-awareness; α = 0.931), and made acceptance composite by combining 3 acceptance related subscales (PMS-acceptance, FFMQ-non-judging, and FFMQ-non-reactivity; α = 0.872). Note that in our pre-registration, we unintentionally included FFMQ-describe in the awareness composite. Analyses using that version of the composite variable are available in the Supplementary Materials and are consistent with the analyses reported here.

**FIGURE 5:**
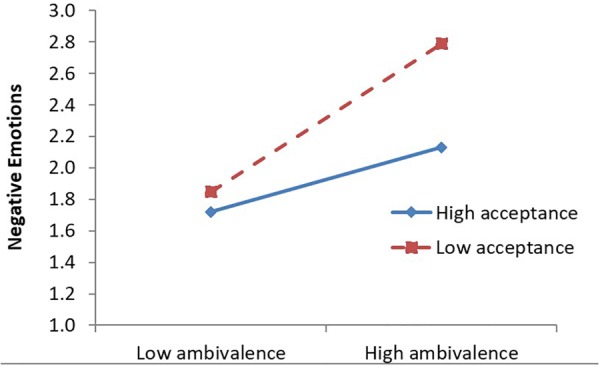
Interaction of ambivalence manipulation and acceptance composite predicting negative affect (Study 5). Graph plotted at ±1*SD* from the sample mean.

In the primary regression analysis predicting SA, there was no interaction between acceptance and the ambivalence manipulation on SA (see Table 2), failing to replicate Study 3. However, when predicting negative affect, acceptance significantly attenuated the effect of the ambivalence manipulation on negative affect and a marginal trend for awareness to enhance the effect of the ambivalence manipulation on negative affect. The acceptance effect on negative affect was parallel to the effect observed on SA in Study 3, such that increases in people’s acceptance of their feelings and thoughts predicted less of a change in negative feelings about Moro bars in response to the ambivalence induction (see Figure 5). The results of the moderation effect of individual full scales or subscales of mindfulness are available in Supplementary Materials.

### Discussion

In this study, which was a very close replication of Study 3, we failed to replicate the primary results obtained in Study 3. Mindfulness did not moderate the effect of ambivalence manipulation on SA. However, we did find some support for our hypotheses on the exploratory dependent measure, negative affect. Specifically, the acceptance composite of mindfulness buffered the effect of ambivalence manipulation on negative affect. This was a pre-registered analysis, which lends some credibility to this finding, but it was explicitly labeled as exploratory in our preregistration. Although it is not immediately clear why we only found the moderation effect of mindfulness on negative affect but not SA, it may be that the negative affect measure is a more direct measure of psychological discomfort than SA. However, this should be interpreted with caution, as the opposite pattern emerged on this dependent measure in Study 4.

## General Discussion

We initially hypothesized that the acceptance component of mindfulness would attenuate the relationship between OA and SA. However, the results of these five studies failed to provide consistent support our hypothesis. In Study 1 the correlational data showed that the acceptance component of mindfulness attenuated the relationship between OA and SA. However, using the same correlational design in Study 2, we did not find support for the buffering effect of acceptance; instead the results suggested that *awareness* predicted a stronger OA–SA relationship. In Study 3, we manipulated ambivalence by introducing a new topic and presenting people with relatively univalent or ambivalent information, and the results showed that acceptance composite of mindfulness buffered the effect of the ambivalence manipulation on SA, consistent with predictions. However, we did not replicate the results in Study 4 when we switched to a different, relatively familiar, attitude object, GM foods. In Study 5, we returned to the same design as Study 3 using a novel topic. Though Study 5 suggested that acceptance composite buffered the effect of ambivalence manipulation on negative affect, we did not replicate the initially observed buffering effect on SA.

It is worth noting that the studies conducted vary in a number of dimensions, though there appears to be variability in the effects both within and across these dimensions. Some studies used measured OA as the predictor (Studies 1 and 2) whereas others used manipulated OA (Studies 3–5). Some studies used existing topics (Studies 1, 2, and 4), whereas others used a novel topic (Studies 3 and 5). These procedural variations do not appear to map onto the strength or direction of the effects observed. Due to the inconsistent findings across all 5 studies, we ran a mini fixed-effects meta-analysis to combine the moderation effect of the awareness and acceptance composites on the OA–SA relationship for Studies 1 and 2 and the effect of the ambivalence manipulation on SA for Studies 3–5. For each study, we used the *t*-value of the interaction term to calculate the effect size in the correlation metric. The results of the meta-analysis suggested that we failed to find support for our predictions (for acceptance composite: *r* = -0.036, 95% CI [-0.087; 0.022]; for awareness composite: *r* = 0.044, 95% CI [-0.010; 0.099]).

So, what might explain the inconsistent effects? First, it may be that the moderation effect of trait mindfulness or trait acceptance is relatively weak, and we were merely observing the variability characteristic of a weaker effect. Second, it may be that the predicted effect was real, but that we did not create optimal conditions to test the effect. For example, perhaps a relatively personally relevant, but still novel topic would create more optimal conditions to examine the effects of acceptance. That way, the importance of the topic might lead the ambivalent information to have greater potential to induce SA or psychological discomfort, and thus create conditions under which acceptance might be relevant, while the novelty of the topic would allow for the potential to circumvent attitude defense processes that might overshadow the effects we have tried to examine. Another possibility, is that mindfulness does not always inhibit automatic responses, and the likelihood that it does may depend on a person’s motivations, such as whether individuals think the automatic response (i.e., the SA and negative affect that follows from OA) is undesirable and not helpful to achieve their goals. That is, it might be possible that mindfulness inhibits automatic responding only when acting in default and habitual ways is maladaptive, such as when it prevents people from reaching their goals. So, the optimal conditions for examining the predicted effects might be ones in which SA is not an adaptive response to OA.

Third, there could be more variability in the effects of mindfulness than is often acknowledged. In support of this possibility, in the work examining the main effect of mindfulness on ambivalence (e.g., Haddock et al., 2017), effects varied in significance and magnitude both across and within the specific mindfulness measures and across and within specific measures of ambivalence employed in the research. Fourth, it is possible that the logic behind the hypothesis was incorrect, either because of poor inference based on published findings or the inferences themselves may have been reasonable, but the published findings on which they were based might not be as robust as they appear on the surface. In support of the latter possibility, concerns have been raised about selective reporting of results in the mindfulness literature (Coronado-Montoya et al., 2016) as well as methodological weaknesses and conceptual ambiguities in studies examining mindfulness (e.g., Van Dam et al., 2017). Indeed, these concerns with past literature are one reason we sought to publish the current findings even though firm conclusions were not possible based on our data.

It is also important to be aware of the variability in the conceptualization and measurement of mindfulness. The conceptualization of mindfulness differs across researchers, with some conceptualizing mindfulness as one factor (e.g., Brown and Ryan, 2003), as two factors (e.g., Bishop et al., 2004), or as five factors (e.g., Baer et al., 2006). Given that individual measures have been criticized on a range of measure construction and psychometric grounds (e.g., Park et al., 2013; Van Dam et al., 2017), we attempted to use a conceptual approach for identifying key aspects of mindfulness (i.e., awareness and acceptance) and to measure these key aspects across several measures. Still, the composites we created are constrained by the measures we used to create them. For example, both the FFMQ and PMS subscales we used to indicate present-moment awareness contain some items that focus on external awareness as well as some items that focus on internal awareness, so they may not best represent the internal awareness (i.e., metacognitive awareness) we sought to examine. In several studies, we also included a meta-awareness scale from an in-development measure (Naragon-Gainey and DeMarree, 2019) that focuses more exclusively on internal awareness, but as this measure has not been fully validated, results should be interpreted with caution. We provide the full analyses of each mindfulness subscale in the Supplementary Materials.

One potential way to address problems with mindfulness measures (e.g., Van Dam et al., 2017) would be to directly manipulate mindfulness or acceptance. Indeed, some recent work has even attempted to separately manipulate awareness and acceptance aspects of mindfulness (e.g., Rahl et al., 2017 did so in a 3-session training). However, past research suggested that acceptance takes longer time to develop than awareness composite of mindfulness (Baer et al., 2012; Desbordes et al., 2015). For a novice meditator starting any sort of training, there is some work suggesting that shifts in awareness are likely to come before shifts in acceptance (Baer et al., 2012; Desbordes et al., 2015), with the latter emerging only with extended training (e.g., 3 weeks in Baer et al., 2012). That is, single session mindfulness interventions are unlikely to affect the acceptance skills that we were hypothesizing would drive the effects. In sum, though direct manipulation of mindfulness or acceptance has the potential to avoid some problems of mindfulness measures and has the potential to allow for the independent manipulation of awareness and acceptance, it may not be feasible to do in the context of a short-term intervention.

Ultimately, we did not reach a solid conclusion regarding whether mindfulness may moderate the relationship between OA and SA. We think this research still makes a valuable contribution. First, in Studies 1 and 2, we replicated earlier findings of Haddock et al. (2017; see also Dummel, 2018) suggesting that mindfulness predicts lower mean levels of both OA and SA. Further, we even obtained evidence consistent with one of the mechanisms that Haddock et al. (2017) hypothesized might be responsible for this effect, reductions in attitudinal discrepancies (see Footnote 2). Second, we treated mindfulness as metacognitive factor that can affect people’s awareness of and appraisal (i.e., acceptance) toward their inner experience. Although the results of this approach were inconsistent when examining ambivalent attitudes predicting ambivalent experience, this general approach may be worth examining in the attitudes and other domains. It is possible that these core metacognitive factors – awareness and acceptance – are useful in understanding core social psychological concepts such as attitudes, information processing, and decision making processes, as these concepts are already lending insights into domains of emotion regulation, well-being, and mental health (e.g., Coffey et al., 2010; Lindsay and Creswell, 2017). Critically, we think it is important to further explore the potential independent effects of different components of mindfulness, which can help to make more clear predictions of the effect of mindfulness and provide insight into the effects of mindfulness and the mechanisms by which these effects occur.

## Ethics Statement

All studies were carried out in accordance with the recommendations of ‘the University at Buffalo Institutional Review Board’ with informed consent from all subjects. All subjects provided consent in accordance with the Declaration of Helsinki. The protocol was approved by the University at Buffalo Institutional Review Board.

## Author Contributions

JW developed the idea. JW and KD designed the studies. JW conducted and analyzed the studies with feedback from KD. JW wrote the manuscript with feedback and edits from KD.

## Conflict of Interest Statement

The authors declare that the research was conducted in the absence of any commercial or financial relationships that could be construed as a potential conflict of interest.

## Footnotes

1 Note, our measures in this study differ from Haddock et al. (2017) because this study was conducted shortly before that paper was published.

2 In addition to the measures described, Study 2 also included measures of actual and desired attitudes. We found that both awareness and acceptance predicted smaller discrepancies between actual and desired attitudes [for awareness: *B* = -0.113, *SE* = 0.046, *t*(205.86) = -2.468, *p* = 0.014; for acceptance: *B* = -0.208, *SE* = 0.054, *t*(205.55) = -3.857, *p* < 0.001]. This finding is consistent with Haddock et al. (2017)’s argument that one reason mindfulness might decrease ambivalence is because it promotes actual-desired attitude discrepancy reduction, and actual-desired attitude discrepancies are one important antecedent of subjective ambivalence (DeMarree et al., 2014). Other measures unrelated to the main hypotheses (e.g., need to evaluate, need for closure, ambivalence frequency, ambivalent comfort, reaction to ambivalence, perceived importance of the issues, and information interest) were also assessed, but are not presented in the main text. However, the full description of these measures and the analyses of these measures can be found in the Supplementary Materials.

3 Measures unrelated to the focal hypothesis are not presented in text (e.g., importance of the issue, certainty and confidence of one’s attitudes toward the issue, the number of arguments one can recall, information interest, and ambivalence coping). A full description of these materials is available in the Supplementary Materials.

4 Measures unrelated to the main hypothesis are not presented in the main text [e.g., importance of the issue, certainty and confidence of one’s attitudes toward the issue, and the number of arguments one can recall, forced choice of supporting or against GM foods, information interest, ambivalence coping, and self-construal scale (Singelis, 1994)], but are described in detail in the Supplementary Materials.

5 https://aspredicted.org/sk5fi.pdf
