# Supplementary material for: An Examination of Whether Mindfulness Can Predict the Relationship Between Objective and Subjective Attitudinal Ambivalence
Source: Front Psychol. 2019 Apr 24;10:854. doi: 10.3389/fpsyg.2019.00854 (PMC6491762; doi:10.3389/fpsyg.2019.00854)
Supplement: Supplementary file 1 [file Table_1.DOCX]

**Study 1 Supplemental Analyses**

Table S1: Correlations between mindfulness measures.

|  | 1 | 2 | 3 |
| --- | --- | --- | --- |
| 1. PMS-awareness |  |  |  |
| 1. PMS-acceptance | -.438^***^ |  |  |
| 1. EQ | .300^**^ | .088 |  |
| 1. CFQ | -.119 | .593^***^ | .184 |

Note. * p < .05, **p < .01, **p <.001

Table S2: Results of multilevel regression analyses examining the main effect of individual mindfulness measures on OA.

|  | Parameter | *b* | *SE* | *t* | *p* |
| --- | --- | --- | --- | --- | --- |
| Model 1 | PMS | -0.112 | 0.051 | -2.17 | .032 |
| Model 2 | PMS-awareness | -0.130 | 0.057 | -2.266 | .025 |
|  | PMS-acceptance | -0.086 | 0.057 | -1.502 | .136 |
| Model 3 | EQ | -0.098 | 0.052 | -1.895 | .061 |
| Model 4 | CFQ | -0.118 | 0.051 | -2.294 | .024 |

Notes. OA = Objective Ambivalence, PMS = Philadelphia Mindfulness Scale, EQ = Experience Questionnaire, CFQ = Cognitive Fusion Questionnaire. All predictors were standardized.

Table S3: Results of multilevel regression analyses examining the main effect of individual mindfulness measures on SA.

|  | Parameter | *b* | *SE* | *t* | *p* |
| --- | --- | --- | --- | --- | --- |
| Model 1 | PMS | -0.215 | 0.119 | -1.805 | .074 |
| Model 2 | PMS-awareness | -0.232 | 0.133 | -1.744 | .084 |
|  | PMS-acceptance | -0.180 | 0.133 | -1.356 | .178 |
| Model 3 | EQ | -0.278 | 0.118 | -2.364 | .020 |
| Model 4 | CFQ | -0.213 | 0.119 | -1.791 | .076 |

Notes. SA = Subjective Ambivalence, PMS = Philadelphia Mindfulness Scale, EQ = Experience Questionnaire, CFQ = Cognitive Fusion Questionnaire. All predictors were standardized.

Table S4: Results of multilevel regression analyses examining the interaction effects of mindfulness and OA in predicting SA and associated simple slopes of OA on SA at high and low levels of each mindfulness subscale.

|  |  | Interaction effect | | | |  | OA at low mind | |  | OA at high mind | |
| --- | --- | --- | --- | --- | --- | --- | --- | --- | --- | --- | --- |
|  | Parameter | *b* | *SE* | *t* | *p* |  | *b* | *SE* |  | *b* | *SE* |
| Model 1 | OA | 0.939 | 0.071 | 13.130 | <0.001 |  |  |  |  |  |  |
|  | PMS | -0.133 | 0.099 | -1.338 | 0.184 |  |  |  |  |  |  |
|  | PMS X OA | -0.138 | 0.068 | -2.030 | 0.045 |  | 1.077 | 0.098 |  | 0.800 | 0.100 |
| Model 2 | OA | 0.930 | 0.070 | 13.336 | <0.001 |  |  |  |  |  |  |
|  | PMS-awareness | -0.138 | 0.111 | -1.239 | 0.218 |  |  |  |  |  |  |
|  | PMS-acceptance | -0.118 | 0.112 | -1.055 | 0.294 |  |  |  |  |  |  |
|  | PMS-awareness X OA | -0.005 | 0.074 | -0.065 | 0.948 |  | 0.932 | 0.102 |  | 0.925 | 0.101 |
|  | PMS-acceptance X OA | -0.235 | 0.074 | -3.166 | 0.002 |  | 1.165 | 0.100 |  | 0.695 | 0.103 |
| Model 3 | OA | 0.939 | 0.073 | 12.931 | <0.001 |  |  |  |  |  |  |
|  | EQ | -0.186 | 0.098 | -1.891 | 0.061 |  |  |  |  |  |  |
|  | EQ X OA | -0.049 | 0.074 | -0.655 | 0.514 |  | 0.988 | 0.105 |  | 0.891 | 0.103 |
| Model 4 | OA | 0.931 | 0.072 | 12.988 | <0.001 |  |  |  |  |  |  |
|  | CFQ | -0.135 | 0.099 | -1.367 | 0.174 |  |  |  |  |  |  |
|  | CFQ X OA | -0.158 | 0.069 | -2.275 | 0.025 |  | 1.089 | 0.097 |  | 0.773 | 0.103 |

Notes. OA = Objective Ambivalence, SA = Subjective Ambivalence, PMS = Philadelphia Mindfulness Scale, EQ = Experience Questionnaire, CFQ = Cognitive Fusion Questionnaire. All simple slopes are significant at *p* < .001. All predictors were standardized.

**Study 2 Additional Measures and Supplemental Analyses**

**Additional Measures**

**Actual attitudes and desired attitudes.** Participants first read the instruction “Sometimes the attitudes we have are different from the attitudes we ideally would like to have and sometimes these attitudes are the same. For your opinion of issue, please indicate the attitude you ACTUALLY have, and the attitude you IDEALLY would like to have using the separate scales provided.” In order to let them know that actual and desired attitudes can be different. Then, they were presented with three items (negative-positive, bad-good, unfavorable-favorable; alpha = 0.968, *M* = 4.639, *SD* = 1.909) in random order and asked to indicate the attitude they **actually** have for each attitude object on a 7-point bipolar scale. After reporting actual attitudes, participants were presented with same three items (alpha = 0.984, *M* = 5.004, *SD* = 1.936) in random order to indicate the attitudes you would **like to** have for each attitude object on a 7-point bipolar scale. The actual-desired attitude discrepancy (*M* = 0.710, *SD* = 1.156) was the absolute value of subtracting the mean of three actual attitude items from the mean of three desired attitude items.

**Importance of the issue.** Two items (adapted from Boninger, Krosnick, & Berent, 1995) measure the extent to which participants find each attitude object are important to themselves on a 4 point scale (1 = *not at all*; 4 = *extremely*; *r*(2082) = 0.766, *p* < .001, *M* = 2.209, *SD* = 0.969). The importance of the issue were assessed by the following items: “How important is this issue to you personally.” and “How important do you consider this issue compared to other issues.”.

**General ambivalence experiences.** Three items adapted from Haddock et al. (2017) were used to measure the ambivalence frequency, ambivalence comfort, and reaction to ambivalence. For ambivalence frequency, participants were asked “How often do you feel ambivalent (never, very rarely, occasionally, sometimes, often, very often, always, *M* = 3.970, *SD* = 1.310)?”. For ambivalence comfort, participants were asked **“**When you feel ambivalent, how comfortable do you feel about this sensation, and respond on a 7-point scale (1= extremely uncomfortable; 7 extremely comfortable, *M* = 3.60, *SD* = 1.122).” For reaction to ambivalence, participants were asked, **“**How do you think you react when you are ambivalent”, and respond on a 7-point scale (1 = *extremely negatively*; 7 = *extremely positively*, *M* = 3.97, *SD* = 0.945)”.

**Need to evaluate.** The need to evaluate measure (Jarvis & Petty, 1996) is a 16-item measure for individual differences in the need to have opinions and to evaluate things on a 5-point scale (1 = *extremely unlike me*; 5 = *extremely like me*; alpha = 0.825, *M* = 2.989, *SD* = 0.548). A sample item is “I form opinions about everything.”

**Need for closure-ambiguity.** The ambiguity subscale of need for closure measure (Webster & Kruglanski, 1994) is a 9-item measure for individual differences in the tolerance of uncertainty and ambiguity on a 6-point scale (1 = *strongly disagree*; 6 = *strongly agree*; alpha = 0.856, *M* = 4.175, *SD* = 0.825). A few sample items are “I don’t like situations that are uncertain.”, and “When I am confused about an important issue, I feel very upset.”.

**Information interest.** For exploratory purposes, we included three items to assess the extent to which people are interested in getting more information about each attitude object (taken from DeMarree et al., 2014). Items gauged participants’ interest in learning more about the topic, getting more information about the topic, and wanting to do something to address any conflicted feelings. All items were answered on a 7-point scale (1 = *not at all*; 7 = *very much*; alpha = 0.954, *M* = 3.240, *SD* = 1.930).

**Supplemental and Exploratory Analyses**

**Information Interest.** We examined whether OA or SA predicted information interest. The results showed that OA did not predict information interest (*B* = 0.003, *SE* = 0.046, *t*(222.33) = 0.064, *p* = .949), but SA positively predicted information interest (*B* = 0.321, *SE* = 0.050, *t*(209.96) = 6.369, *p* < .001). That is, people felt more conflicted feelings were more willing to seek out for more information to resolve their ambivalence. We also explored whether mindfulness may affect people’s interest in resolving ambivalence, so we examined whether mindfulness may interact with OA or SA to predict information interest. However, neither awareness nor acceptance moderated the link between OA (*ps* > .2) or SA (*ps* > .1) and information interest.

Table S5: Correlation between mindfulness measures.

|  | 1 | 2 | 3 | 4 | 5 | 6 |
| --- | --- | --- | --- | --- | --- | --- |
| 1. PMS-awareness |  |  |  |  |  |  |
| 1. PMS-acceptance | -.424^***^ |  |  |  |  |  |
| 1. FFMQ-observe | .715^***^ | -.403^***^ |  |  |  |  |
| 1. FFMQ-describe | .392^***^ | .211^**^ | .196^**^ |  |  |  |
| 1. FFMQ-acting with awareness | -.138^*^ | .429^***^ | -.292^***^ | .248^***^ |  |  |
| 1. FFMQ-nonjudging | -.194^**^ | .617^***^ | -.301^***^ | .284^***^ | .513^***^ |  |
| 1. FFMQ-nonreactivity | .476^***^ | -.133 | .431^***^ | .325^***^ | -.156^*^ | -.031 |

Notes. PMS = Philadelphia Mindfulness Scale, FFMQ = Five Facet Mindfulness Questionnaire.

Table S6: Correlation between mindfulness, ambivalence frequency, ambivalence comfort, and reaction to ambivalence

|  | Ambivalence frequency | Ambivalence comfort | React positively to ambivalence | Need to evaluate | Need for closure - ambiguity |
| --- | --- | --- | --- | --- | --- |
| 1. PMS-awareness | .071 | -.003 | .089 | .133 | .392^***^ |
| 1. PMS-acceptance | -.330^***^ | .119 | .018 | -.083 | -.490^***^ |
| 1. FFMQ-observe | .130 | .060 | .087 | .135 | .357^**^ |
| 1. FFMQ-describe | -.175^*^ | .209^**^ | .200^**^ | .148^*^ | .014 |
| 1. FFMQ-acting with awareness | -.255^***^ | .134 | .078 | -.193^**^ | -.200^**^ |
| 1. FFMQ-nonjudging | -.337^***^ | .217^**^ | .061 | -.123 | -.323^***^ |
| 1. FFMQ- nonreactivity | -.010 | .183^**^ | .172^*^ | -.068 | .106 |
| 1. Awareness composite | .119 | .046 | .111 | .156^*^ | .411^***^ |
| 1. Acceptance composite | -.359^***^ | .215^**^ | .082 | -.163^*^ | -.373^***^ |

Note. PMS = Philadelphia Mindfulness Scale, FFMQ = Five Facet Mindfulness Questionnaire. Awareness composite is the combination of PMS-awareness, FFMQ-Observe. Acceptance composite is the combination of PMS-acceptance, FFMQ-Nonjudging, FFMQ-Nonreactivity.

Table S7: Results of multilevel regression analyses examining the main effect of mindfulness on actual-desired attitude discrepancy.

|  | Parameter | *b* | *SE* | *t* | *p* |
| --- | --- | --- | --- | --- | --- |
| Model 1 | PMS | -0.159 | 0.037 | -4.347 | .000 |
| Model 2 | PMS-awareness | -0.113 | 0.040 | -2.815 | .005 |
|  | PMS-acceptance | -0.182 | 0.040 | -4.525 | .000 |
| Model 3 | FFMQ | -0.161 | 0.037 | -4.377 | .000 |
| Model 4 | FFMQ-Observe | -0.057 | 0.044 | -1.286 | .200 |
|  | FFMQ-Describe | -0.112 | 0.043 | -2.617 | .010 |
|  | FFMQ-Acting with awareness | -0.013 | 0.045 | -0.282 | .778 |
|  | FFMQ-Nonjudging | -0.094 | 0.045 | -2.073 | .039 |
|  | FFMQ-Nonreactivity | 0.009 | 0.043 | 0.212 | .832 |
| Model 5 | Awareness composite | -0.069 | 0.040 | -1.742 | .083 |
|  | Acceptance composite | -0.227 | 0.055 | -4.143 | .000 |

Note. PMS = Philadelphia Mindfulness Scale, FFMQ = Five Facet Mindfulness Questionnaire. Awareness composite is the combination of PMS-awareness, FFMQ-Observe. Acceptance composite is the combination of PMS-acceptance, FFMQ-Nonjudging, FFMQ-Nonreactivity. All predictors were standardized.

Table S8: Results of multilevel regression analyses examining the main effect of mindfulness on OA.

|  | Parameter | *b* | *SE* | *t* | *p* |
| --- | --- | --- | --- | --- | --- |
| Model 1 | PMS | -0.093 | 0.044 | -2.138 | .034 |
| Model 2 | PMS-awareness | -0.136 | 0.048 | -2.854 | .005 |
|  | PMS-acceptance | -0.038 | 0.048 | -0.805 | .421 |
| Model 3 | FFMQ | -0.106 | 0.044 | -2.424 | .016 |
| Model 4 | FFMQ-Observe | -0.030 | 0.052 | -0.576 | .565 |
|  | FFMQ-Describe | -0.052 | 0.050 | -1.036 | .302 |
|  | FFMQ-Acting with awareness | -0.071 | 0.053 | -1.345 | .180 |
|  | FFMQ-Nonjudging | -0.077 | 0.053 | -1.446 | .150 |
|  | FFMQ-Nonreactivity | 0.058 | 0.051 | 1.149 | .252 |

Note. OA = Objective Ambivalence, PMS = Philadelphia Mindfulness Scale, FFMQ = Five Facet Mindfulness Questionnaire. All predictors were standardized.

Table S9: Results of multilevel regression analyses examining the main effect of mindfulness on SA.

|  | Parameter | *b* | *SE* | *t* | *p* |
| --- | --- | --- | --- | --- | --- |
| Model 1 | PMS | -0.214 | 0.069 | -3.085 | .002 |
| Model 2 | PMS-awareness | -0.170 | 0.077 | -2.216 | .028 |
|  | PMS-acceptance | -0.228 | 0.077 | -2.971 | .003 |
| Model 3 | FFMQ | -0.151 | 0.070 | -2.148 | .033 |
| Model 4 | FFMQ-Observe | 0.000 | 0.084 | -0.006 | .995 |
|  | FFMQ-Describe | -0.100 | 0.081 | -1.229 | .221 |
|  | FFMQ-Acting with awareness | -0.072 | 0.085 | -0.847 | .398 |
|  | FFMQ-Nonjudging | -0.130 | 0.086 | -1.514 | .132 |
|  | FFMQ-Nonreactivity | 0.085 | 0.082 | 1.043 | .298 |

Note. SA = Subjective Ambivalence, PMS = Philadelphia Mindfulness Scale, FFMQ = Five Facet Mindfulness Questionnaire. All predictors were standardized.

Table S10: Results of regression analyses examining the interaction effects of mindfulness and ambivalence manipulation in predicting SA and associated simple slopes of condition on SA at high and low levels of each mindfulness subscale.

|  |  | Interaction effect | | | |  | OA at low mind | |  | OA at high mind | |
| --- | --- | --- | --- | --- | --- | --- | --- | --- | --- | --- | --- |
|  | Parameter | *b* | *SE* | *t* | *p* |  | *b* | *SE* |  | *b* | *SE* |
| Model 1 | OA | 0.774 | 0.049 | 15.917 | .000 |  |  |  |  |  |  |
|  | PMS | -0.128 | 0.055 | -2.321 | .021 |  |  |  |  |  |  |
|  | PMS X OA | 0.045 | 0.047 | 0.974 | .331 |  | 0.729 | 0.067 |  | 0.819 | 0.068 |
| Model 2 | OA | 0.776 | 0.049 | 15.980 | .000 |  |  |  |  |  |  |
|  | PMS-awareness | -0.070 | 0.061 | -1.131 | .259 |  |  |  |  |  |  |
|  | PMS-acceptance | -0.168 | 0.062 | -2.735 | .007 |  |  |  |  |  |  |
|  | PMS-awareness X OA | 0.074 | 0.052 | 1.433 | .154 |  | 0.702 | 0.072 |  | 0.851 | 0.070 |
|  | PMS-acceptance X OA | 0.013 | 0.051 | 0.265 | .791 |  | 0.763 | 0.069 |  | 0.790 | 0.072 |
| Model 3 | OA | 0.774 | 0.049 | 15.852 | .000 |  |  |  |  |  |  |
|  | FFMQ | -0.057 | 0.056 | -1.020 | .309 |  |  |  |  |  |  |
|  | FFMQ X OA | 0.068 | 0.046 | 1.474 | .142 |  | 0.705 | 0.067 |  | 0.842 | 0.068 |
| Model 4 | OA | 0.766 | 0.048 | 15.794 | .000 |  |  |  |  |  |  |
|  | FFMQ-Observe | 0.027 | 0.067 | 0.408 | .684 |  |  |  |  |  |  |
|  | FFMQ-Describe | -0.060 | 0.065 | -0.925 | .356 |  |  |  |  |  |  |
|  | FFMQ-Acting with awareness | 0.019 | 0.069 | 0.282 | .778 |  |  |  |  |  |  |
|  | FFMQ-Nonjudging | -0.077 | 0.069 | -1.108 | .269 |  |  |  |  |  |  |
|  | FFMQ-Nonreactivity | 0.029 | 0.067 | 0.435 | .664 |  |  |  |  |  |  |
|  | FFMQ-Observe X OA | 0.061 | 0.056 | 1.094 | .275 |  | 0.704 | 0.073 |  | 0.827 | 0.075 |
|  | FFMQ-Describe X OA | 0.086 | 0.054 | 1.578 | .116 |  | 0.680 | 0.074 |  | 0.851 | 0.072 |
|  | FFMQ-Acting with awareness X OA | 0.088 | 0.056 | 1.563 | .120 |  | 0.677 | 0.073 |  | 0.854 | 0.076 |
|  | FFMQ-Nonjudging X OA | -0.110 | 0.058 | -1.900 | .059 |  | 0.875 | 0.074 |  | 0.656 | 0.077 |
|  | FFMQ-Nonreactivity X OA | 0.014 | 0.055 | 0.254 | .799 |  | 0.752 | 0.074 |  | 0.779 | 0.072 |

Note. OA = Objective Ambivalence, SA = Subjective Ambivalence, PMS = Philadelphia Mindfulness Scale, FFMQ = Five Facet Mindfulness Questionnaire. All simple slopes are significant, *p* < .001. All predictors were standardized.

**Study 3 Additional Measures and Supplemental Analyses**

**Additional Measures**

**Recalling the pros and cons arguments of Moro bars.** Participants were instructed to recall as much of the information about Moro bars that they read before.

**Importance of the issue.** To measure importance participants completed the same two items used in Study 2 (*r*(278) = .644, p < .001, *M* = 2.130, *SD* = 1.333).

**Attitude certainty.** Participants indicated their attitude certainty using two items measuring how certain and how confident they were in their attitude toward each Moro Bars on a 7-point scale (1 = *not at all*; 7 = *very much*; *r*(278) = .869, p < .001, *M* = 5.171, *SD* = 1.696).

**Information interest.** Information interest was measured using three items similar to those used in Study 2 (alpha = 0.899, *M* = 2.764, *SD* = 1.643). In addition, we also asked more specific about information interest toward positive and negative information about Moro bars. For positive information interest, there were two items (*r*(278) = .864, p < .001, *M* = 3.453, *SD* = 1.782) asking “To what extent would you like to read positive information about/ read information in support of Moro bars” on a 7 point scale (1 = *not at all*; 7 = *very much*). For negative information interest, there were two comparably worded items (*r*(278) = .888, p < .001, *M* = 3.372, *SD* = 1.806). Past research suggest that people holding ambivalent attitudes showed biased information processing. That is, they tended to pay more attention on pro-attitudinal information and avoid counter-attitudinal information (Clark, Wegener, & Fabrigar, 2008). So, we also examined whether mindfulness can influence biased information processing.

**Ambivalence Coping.** We attempted to measure strategies by which people might cope with ambivalence, using items adapted from Nohlen et al. (2015). Specifically, participants were told that “Before you make a decision for or against Moro Bars, please choose the activity you prefer to do in the next section of the study”. They were presented with two options “writing down the pros and cons of the issue” which would be a more problem-focused way to deal with ambivalence or the other option is “describe the route from your house to the university” which would be a less problem-focus and more of a distraction from ambivalence (34.2% participants chose problem-focused option, and 65.8% participants chose distraction).

**Supplemental and Exploratory analyses**

We first examined whether SA predicted information interest. SA was positively correlated with information interest in this sample (*r*(278) = .170, *p* = .005). That is, people who felt more conflicted feelings were more interested in obtaining more information to resolve their ambivalence. We also explored whether mindfulness may affect how people deal with ambivalence by examining whether mindfulness interacted with SA to predict information interest. However, neither awareness nor acceptance moderated the link between SA and information interest (*ps* > .2).

We next examined whether increases in SA would predict greater preference for pro-attitudinal information, where pro-attitudinal was defined as information consistent with their “dominant” side (i.e., the greater of their positive and negative evaluation). To do this, we examine whether SA interacted with dominant reaction (if positive reaction is stronger than negative reaction, then it was coded as 1; if positive and negative reaction is equally strong, then it was coded as 0; if negative reaction is stronger than positive reaction, then it was coded as -1) to predict seeking for positive information and negative information separately. However, there was no interaction effect on positive or negative information seeking (*ps* > .3).

We further examined whether awareness or acceptance composite moderate this interaction between SA and dominant reaction on positive and negative information interest. However, only the three-way interaction between dominant reaction, SA, and awareness composite is significant (*B* = 0.347, *SE* = 0.159, *t*(266) = 2.187, *p* = .03). That is, people with high awareness are more likely to seek negative information when the dominant reaction is positive and they feel high SA, which did not make sense at all.

Finally, we also examined whether SA predicted people’s ambivalence coping strategies. SA marginally positively predicted the likelihood of choosing problem-focus approach over distraction (*B* = 0.158, *SE* = 0.083, *Wald* = 3.632, *p* = .057). However, acceptance and awareness didn’t moderate the relationship between SA and ambivalence-reduction choice (*ps* > .11).

Table S11: Descriptive statistics and correlations between mindfulness and ambivalence.

|  | Mean | SD | 1 | 2 | 3 | 4 | 5 | 6 | 7 | 8 | 9 | 10 | 11 |
| --- | --- | --- | --- | --- | --- | --- | --- | --- | --- | --- | --- | --- | --- |
| 1. PMS | 3.19 | 0.40 |  |  |  |  |  |  |  |  |  |  |  |
| 1. PMS-awareness | 3.72 | 0.54 | .52^***^ |  |  |  |  |  |  |  |  |  |  |
| 1. PMS-acceptance | 2.66 | 0.69 | .74^***^ | -.19^**^ |  |  |  |  |  |  |  |  |  |
| 1. FFMQ | 3.21 | 0.41 | .69^***^ | .39*^**^ | .49^***^ |  |  |  |  |  |  |  |  |
| 1. FFMQ-Observe | 3.39 | 0.66 | .25^***^ | .66^***^ | -.23^**^ | .31^***^ |  |  |  |  |  |  |  |
| 1. FFMQ-Describe | 3.29 | 0.70 | .50^***^ | .29^***^ | .34^***^ | .74^***^ | .05 |  |  |  |  |  |  |
| 1. FFMQ-Acting with awareness | 3.09 | 0.70 | .43^***^ | .05 | .46^***^ | .67^***^ | -.11 | .42^***^ |  |  |  |  |  |
| 1. FFMQ-Nonjudging | 3.18 | 0.78 | .53^***^ | -.03 | .64^***^ | .69^***^ | -.17^**^ | .40^***^ | .47^***^ |  |  |  |  |
| 1. FFMQ-Nonreactivity | 3.08 | 0.54 | .36^***^ | .30^***^ | .18^**^ | .60^***^ | .27^***^ | .34^***^ | .15^*^ | .24^***^ |  |  |  |
| 1. Meta-awareness | 5.51 | 0.88 | .42^***^ | .65^***^ | -.03 | .39^***^ | .45^***^ | .29^***^ | .13^*^ | .07 | .31^***^ |  |  |
| 1. OA | 1.56 | 1.82 | -.03 | -.12^*^ | .06 | .02 | -.02 | -.07 | .15^*^ | .01 | -.03 | -.16^**^ |  |
| 1. SA | 2.95 | 1.53 | -.12 | -.09 | -.06 | -.12^*^ | -.02 | -.10 | -.02 | -.13^*^ | -.012 | -.17^**^ | .48^***^ |

Note. PMS = Philadelphia Mindfulness Scale, FFMQ = Five Facet Mindfulness Questionnaire. OA = Objective Ambivalence. SA = Subjective Ambivalence. **p* < .05, ***p* < .01, ****p* < .001.

Table S12: Results of regression analyses examining the interaction effects of mindfulness and ambivalence manipulation in predicting SA and associated simple slopes of condition on SA at high and low levels of each mindfulness subscale.

|  |  | Interaction effect | | | |  | cond at low mind | |  | cond at high mind | |
| --- | --- | --- | --- | --- | --- | --- | --- | --- | --- | --- | --- |
|  | Parameter | *b* | *SE* | *t* | *p* |  | *b* | *SE* |  | *b* | *SE* |
| Model 1 | Condition | 0.909 | 0.177 | 5.138 | .000 |  |  |  |  |  |  |
|  | PMS | -0.161 | 0.109 | -1.476 | .141 |  |  |  |  |  |  |
|  | Condition x PMS | -0.160 | 0.184 | -0.867 | .387 |  | 1.069*** | 0.260 |  | 0.750** | 0.250 |
| Model 2 | Condition | 0.913 | 0.177 | 5.143 | .000 |  |  |  |  |  |  |
|  | PMS-awareness | -0.206 | 0.119 | -1.734 | .084 |  |  |  |  |  |  |
|  | PMS-acceptance | -0.083 | 0.109 | -0.762 | .447 |  |  |  |  |  |  |
|  | Condition x PMS-awareness | 0.061 | 0.18 | 0.338 | .735 |  | 0.852** | 0.254 |  | 0.974*** | 0.251 |
|  | Condition x PMS-acceptance | -0.268 | 0.191 | -1.406 | .161 |  | 1.181*** | 0.268 |  | 0.644* | 0.254 |
| Model 3 | Condition | 0.931 | 0.176 | 5.282 | .000 |  |  |  |  |  |  |
|  | FFMQ | -0.110 | 0.117 | -0.937 | .350 |  |  |  |  |  |  |
|  | Condition x FFMQ | -0.301 | 0.176 | -1.707 | .089 |  | 1.232*** | 0.253 |  | 0.630* | 0.246 |
| Model 4 | Condition | 0.896 | 0.177 | 5.070 | .000 |  |  |  |  |  |  |
|  | FFMQ-Observe | -0.136 | 0.128 | -1.065 | .288 |  |  |  |  |  |  |
|  | FFMQ-Describe | -0.190 | 0.129 | -1.469 | .143 |  |  |  |  |  |  |
|  | FFMQ-Acting with awareness | 0.101 | 0.125 | 0.814 | .416 |  |  |  |  |  |  |
|  | FFMQ-Nonjudging | -0.027 | 0.136 | -0.199 | .842 |  |  |  |  |  |  |
|  | FFMQ-Nonreactivity | 0.022 | 0.134 | 0.162 | .871 |  |  |  |  |  |  |
|  | Condition x FFMQ-Observe | 0.207 | 0.188 | 1.096 | .274 |  | 0.690** | 0.261 |  | 1.103*** | 0.256 |
|  | Condition x FFMQ-Describe | 0.261 | 0.215 | 1.212 | .226 |  | 0.635* | 0.277 |  | 1.158*** | 0.280 |
|  | Condition x FFMQ-Acting with awareness | -0.111 | 0.225 | -0.496 | .620 |  | 1.008** | 0.294 |  | 0.785** | 0.277 |
|  | Condition x FFMQ-Nonjudging | -0.403 | 0.218 | -1.849 | .066 |  | 1.299*** | 0.277 |  | 0.494 | 0.284 |
|  | Condition x FFMQ-Nonreactivity | -0.366 | 0.197 | -1.863 | .064 |  | 1.263*** | 0.263 |  | 0.530* | 0.266 |
| Model 5 | Condition | 0.848 | 0.176 | 4.821 | .000 |  |  |  |  |  |  |
|  | Meta-awareness | -0.204 | 0.114 | -1.789 | .075 |  |  |  |  |  |  |
|  | Condition x Meta-awareness | -0.085 | 0.178 | -0.479 | .632 |  | 0.933*** | 0.249 |  | 0.763** | 0.251 |

Note. PMS = Philadelphia Mindfulness Scale, FFMQ = Five Facet Mindfulness Questionnaire. SA = Subjective Ambivalence. All predictors were standardized. **p* < .05, ***p* < .01, ****p* < .001.

**Study 4 Additional Measures and Supplemental Analyses**

**Additional Measures**

**Information interest.** Information interest was assessed using three items similar to Study 3 (alpha = 0.901, *M* = 3.827, *SD* = 1.572) measuring general information interest in GM foods. Two items measuring interest in positive information (r(353) = .794, p < .001, *M* = 3.805, *SD* = 1.642) and negative information (r(353) = .771, p < .001, *M* = 3.908, *SD* = 1.689) for GM foods were also used in this study.

**Ambivalence coping.** We adapted the dichotomous choice measure of ambivalence coping strategies in this study to assess interest in each strategy in a continuous manner. Specifically, participants rated the extent to which they would like to do each of the two activities—writing down the pros and cons of the issue (problem-focused choice, *M* = 3.41, *SD* = 1.742) and describing the route from your house to the university (avoidance choice, *M* = 3.54, *SD* = 1.873) on a seven-point scale (1=*not at all*; 7= *very much*).

**Importance of the issue.** Same two items (r(354) = .562, p < .001, *M* = 3.617, *SD* = 1.442) from Study 2.

**Certainty and confidence in the attitude toward the issue.** Same items (1 = *not at all*; 7 = *very much*; *r*(354) = .756 , p < .001, *M* = 4.353, *SD* = 1.539). from Study 3.

**Forced choice of attitude.** Participants were forced to decide whether their general attitude toward GM foods is in support of GM foods (56.4%) or against GM foods (43.6%).

**Recall the pros and cons argument of GM foods.** Same as Study 3.

**Self-construal scale.** 15 items (Singelis, 1994) measure the extent to which one define oneself by one’s own uniqueness, which is independent self-construal (alpha = 0.851, *M* = 4.707, *SD* = 0.775) and 15 item measure the extent to which one define oneself by one’s relationship and connection to others which is interdependent self-construal (alpha = 0.838, *M* = 4.601, *SD* = 0.735) on a 7-point scale (1 = *strongly disagree*; 7 = *strongly agree*).

**Supplemental and Exploratory analyses**

We first examined whether SA predicted information interest. The results showed that SA is positively correlated with information interest (*r*(354) = .135, *p* = .011). Consistent with previous studies, people felt more conflicted feelings were more willing to seek out for more information. We also explored whether mindfulness may affect how people deal with ambivalence, so we examined whether mindfulness may interact with SA to predict information interest. However, neither awareness nor acceptance moderated the link between SA and information interest (*ps* > .3).

We then examined whether people with high SA would prefer pro-attitudinal information more, so we examine whether SA interacted with dominant reaction to predict seeking for positive information and negative information separately. However, there was no interaction effect on positive or negative information interest (*ps* > .4). The three-way interaction between awareness or acceptance, SA, and dominant reaction did not predict positive or negative information interest (*ps* > .1).

We also examined whether SA predicted how people deal with ambivalence, and the results showed that SA correlated positively with interest in both problem-focused (*r*(354) = .143, *p* = .007) and avoidance strategies (*r*(354) = .189, *p* < .001). However, acceptance and awareness didn’t moderate the relationship between SA and ambivalence-reduction choice (*ps* > .11).

Table S13: Descriptive statistics and correlations between mindfulness and ambivalence.

|  | Mean | SD | 1 | 2 | 3 | 4 | 5 | 6 | 7 | 8 | 9 | 10 | 11 | 12 |
| --- | --- | --- | --- | --- | --- | --- | --- | --- | --- | --- | --- | --- | --- | --- |
| 1. PMS | 3.13 | 0.36 |  |  |  |  |  |  |  |  |  |  |  |  |
| 1. PMS-awareness | 3.56 | 0.60 | .50^***^ |  |  |  |  |  |  |  |  |  |  |  |
| 1. PMS-acceptance | 2.69 | 0.67 | .63^***^ | -.35^***^ |  |  |  |  |  |  |  |  |  |  |
| 1. FFMQ | 3.09 | 0.32 | .69^***^ | .45^***^ | .35^***^ |  |  |  |  |  |  |  |  |  |
| 1. FFMQ-Observe | 3.34 | 0.66 | .27^***^ | .61^***^ | -.25^***^ | .41^***^ |  |  |  |  |  |  |  |  |
| 1. FFMQ-Describe | 3.13 | 0.58 | .41^***^ | .40^***^ | .08 | .70^***^ | .25^***^ |  |  |  |  |  |  |  |
| 1. FFMQ-Acting with awareness | 2.90 | 0.62 | .27^***^ | -.10 | .38^***^ | .44^***^ | -.25^***^ | .08 |  |  |  |  |  |  |
| 1. FFMQ-Nonjudging | 2.96 | 0.76 | .49^***^ | -.07 | .58^***^ | .56^***^ | -.29^***^ | .19^***^ | .45^***^ |  |  |  |  |  |
| 1. FFMQ-Nonreactivity | 3.15 | 0.57 | .29^***^ | .40^***^ | -.04 | .42^***^ | .46^***^ | .32^***^ | -.29^***^ | -.19^***^ |  |  |  |  |
| 1. Meta-awareness | 5.23 | 1.03 | .37^***^ | .72^***^ | -.24^***^ | .36^***^ | .51^***^ | .30^***^ | -.09 | -.10 | .39^***^ |  |  |  |
| 1. OA | 2.06 | 1.73 | -.05 | -.06 | .00 | -.07 | .05 | -.07 | -.07 | -.08 | .02 | -.05 |  |  |
| 1. SA | 3.85 | 1.43 | .03 | .11^*^ | -.07 | .07 | .15^**^ | .03 | -.04 | -.03 | .07 | .09 | .55^***^ |  |
| 1. Negative emotions | 2.62 | 1.41 | -.17^**^ | -.10 | -.09 | -.12^*^ | .07 | .01 | -.20^***^ | -.21^***^ | .08 | -.06 | .16^**^ | .21^***^ |

Note. PMS = Philadelphia Mindfulness Scale, FFMQ = Five Facet Mindfulness Questionnaire. OA = Objective Ambivalence. SA = Subjective Ambivalence. **p* < .05, ***p* < .01, ****p* < .001

Table S14: Results of regression analyses examining the interaction effects of mindfulness and ambivalence manipulation in predicting SA and associated simple slopes of condition on SA at high and low levels of each mindfulness variable.

|  |  | Interaction effect | | | |  | cond at low mind | |  | cond at high mind | |
| --- | --- | --- | --- | --- | --- | --- | --- | --- | --- | --- | --- |
|  | Parameter | *b* | *SE* | *t* | *p* |  | *b* | *SE* |  | *b* | *SE* |
| Model 1 | Condition | 0.099 | 0.152 | 0.652 | .515 |  |  |  |  |  |  |
|  | PMS | -0.126 | 0.101 | -1.251 | .212 |  |  |  |  |  |  |
|  | Condition x PMS | 0.383 | 0.154 | 2.492 | .013 |  | -0.284 | 0.215 |  | 0.482* | 0.217 |
| Model 2 | Condition | 0.097 | 0.151 | 0.638 | .524 |  |  |  |  |  |  |
|  | PMS-awareness | -0.001 | 0.108 | -0.005 | .996 |  |  |  |  |  |  |
|  | PMS-acceptance | -0.202 | 0.109 | -1.854 | .065 |  |  |  |  |  |  |
|  | Condition x PMS-awareness | 0.332 | 0.163 | 2.040 | .042 |  | -0.236 | 0.221 |  | 0.429 | 0.223 |
|  | Condition x PMS-acceptance | 0.349 | 0.163 | 2.143 | .033 |  | -0.252 | 0.221 |  | 0.445* | 0.223 |
| Model 3 | Condition | 0.103 | 0.153 | 0.674 | .501 |  |  |  |  |  |  |
|  | FFMQ | 0.048 | 0.098 | 0.490 | .625 |  |  |  |  |  |  |
|  | Condition x FFMQ | 0.126 | 0.157 | 0.806 | .421 |  | -0.023 | 0.217 |  | 0.229 | 0.221 |
| Model 4 | Condition | 0.091 | 0.153 | 0.598 | .550 |  |  |  |  |  |  |
|  | FFMQ-Observe | 0.176 | 0.123 | 1.430 | .154 |  |  |  |  |  |  |
|  | FFMQ-Describe | 0.035 | 0.111 | 0.318 | .751 |  |  |  |  |  |  |
|  | FFMQ-Acting with awareness | -0.006 | 0.126 | -0.050 | .960 |  |  |  |  |  |  |
|  | FFMQ-Nonjudging | -0.121 | 0.121 | -1.004 | .316 |  |  |  |  |  |  |
|  | FFMQ-Nonreactivity | 0.023 | 0.126 | 0.180 | .857 |  |  |  |  |  |  |
|  | Condition x FFMQ-Observe | 0.117 | 0.183 | 0.640 | .523 |  | -0.026 | 0.239 |  | 0.208 | 0.238 |
|  | Condition x FFMQ-Describe | -0.138 | 0.175 | -0.790 | .430 |  | 0.230 | 0.232 |  | -0.047 | 0.233 |
|  | Condition x FFMQ-Acting with awareness | -0.033 | 0.178 | -0.187 | .852 |  | 0.125 | 0.234 |  | 0.058 | 0.234 |
|  | Condition x FFMQ-Nonjudging | 0.366 | 0.183 | 2.004 | .046 |  | -0.274 | 0.238 |  | 0.457 | 0.238 |
|  | Condition x FFMQ-Nonreactivity | 0.001 | 0.184 | 0.004 | .996 |  | 0.091 | 0.239 |  | 0.092 | 0.240 |
| Model 5 | Condition | 0.094 | 0.152 | 0.614 | .539 |  |  |  |  |  |  |
|  | Meta-awareness | 0.109 | 0.104 | 1.046 | .296 |  |  |  |  |  |  |
|  | Condition x Meta-awareness | 0.035 | 0.153 | 0.227 | .821 |  | 0.059 | 0.216 |  | 0.128 | 0.216 |

Note. PMS = Philadelphia Mindfulness Scale, FFMQ = Five Facet Mindfulness Questionnaire. OA = Objective Ambivalence. SA = Subjective Ambivalence. All predictors were standardized. **p* < .05.

Table S15: Results of regression analyses examining the interaction effects of mindfulness and ambivalence manipulation in predicting negative emotions and associated simple slopes of condition on negative emotions at high and low levels of each mindfulness variable.

|  |  | Interaction effect | | | |  | cond at low mind | |  | cond at high mind | |
| --- | --- | --- | --- | --- | --- | --- | --- | --- | --- | --- | --- |
|  | Parameter | *b* | *SE* | *t* | *p* |  | *b* | *SE* |  | *b* | *SE* |
| Model 1 | Condition | 0.398 | 0.147 | 2.711 | .007 |  |  |  |  |  |  |
|  | PMS | -0.257 | 0.097 | -2.647 | .008 |  |  |  |  |  |  |
|  | Condition x PMS | 0.087 | 0.148 | 0.585 | .559 |  | 0.311 | 0.207 |  | 0.485* | 0.210 |
| Model 2 | Condition | 0.398 | 0.147 | 2.713 | .007 |  |  |  |  |  |  |
|  | PMS-awareness | -0.159 | 0.105 | -1.514 | .131 |  |  |  |  |  |  |
|  | PMS-acceptance | -0.282 | 0.105 | -2.677 | .008 |  |  |  |  |  |  |
|  | Condition x PMS-awareness | -0.074 | 0.158 | -0.467 | .641 |  | 0.472* | 0.215 |  | 0.324 | 0.217 |
|  | Condition x PMS-acceptance | 0.194 | 0.158 | 1.227 | .221 |  | 0.204 | 0.214 |  | 0.592** | 0.217 |
| Model 3 | Condition | 0.408 | 0.148 | 2.762 | .006 |  |  |  |  |  |  |
|  | FFMQ | -0.204 | 0.094 | -2.160 | .031 |  |  |  |  |  |  |
|  | Condition x FFMQ | 0.110 | 0.151 | 0.724 | .469 |  | 0.298 | 0.210 |  | 0.517* | 0.213 |
| Model 4 | Condition | 0.366 | 0.143 | 2.569 | .011 |  |  |  |  |  |  |
|  | FFMQ-Observe | -0.050 | 0.115 | -0.434 | .665 |  |  |  |  |  |  |
|  | FFMQ-Describe | 0.234 | 0.104 | 2.262 | .024 |  |  |  |  |  |  |
|  | FFMQ-Acting with awareness | -0.122 | 0.118 | -1.040 | .299 |  |  |  |  |  |  |
|  | FFMQ-Nonjudging | -0.535 | 0.113 | -4.745 | .000 |  |  |  |  |  |  |
|  | FFMQ-Nonreactivity | -0.057 | 0.118 | -0.482 | .630 |  |  |  |  |  |  |
|  | Condition x FFMQ-Observe | 0.099 | 0.171 | 0.581 | .562 |  | 0.267 | 0.223 |  | 0.466* | 0.222 |
|  | Condition x FFMQ-Describe | -0.383 | 0.164 | -2.342 | .020 |  | 0.750** | 0.217 |  | -0.017 | 0.217 |
|  | Condition x FFMQ-Acting with awareness | -0.130 | 0.166 | -0.787 | .432 |  | 0.497* | 0.219 |  | 0.236 | 0.219 |
|  | Condition x FFMQ-Nonjudging | 0.695 | 0.170 | 4.077 | .000 |  | -0.328 | 0.222 |  | 1.061*** | 0.223 |
|  | Condition x FFMQ-Nonreactivity | 0.155 | 0.172 | 0.903 | .367 |  | 0.211 | 0.223 |  | 0.522* | 0.224 |
| Model 5 | Condition | 0.422 | 0.148 | 2.851 | 0.005 |  |  |  |  |  |  |
|  | Meta-awareness | -0.026 | 0.101 | -0.257 | 0.797 |  |  |  |  |  |  |
|  | Condition x Meta-awareness | -0.130 | 0.148 | -0.878 | 0.380 |  | 0.552** | 0.209 |  | 0.291 | 0.210 |

Note. PMS = Philadelphia Mindfulness Scale, FFMQ = Five Facet Mindfulness Questionnaire. OA = Objective Ambivalence. SA = Subjective Ambivalence. All predictors were standardized. **p* < .05, ***p* < .01, ****p* < .001

**Study 5 Supplemental Analyses**

Table S16: Descriptive statistics and correlations between mindfulness and ambivalence.

|  | Mean | SD | 1 | 2 | 3 | 4 | 5 | 6 | 7 | 8 | 9 | 10 | 11 | 12 |
| --- | --- | --- | --- | --- | --- | --- | --- | --- | --- | --- | --- | --- | --- | --- |
| 1. PMS | 3.12 | 0.42 |  |  |  |  |  |  |  |  |  |  |  |  |
| 1. PMS-awareness | 3.65 | 0.63 | .57^***^ |  |  |  |  |  |  |  |  |  |  |  |
| 1. PMS-acceptance | 2.60 | 0.70 | .68^***^ | -.22^***^ |  |  |  |  |  |  |  |  |  |  |
| 1. FFMQ | 3.13 | 0.43 | .72^***^ | .46^***^ | .45^***^ |  |  |  |  |  |  |  |  |  |
| 1. FFMQ-Observe | 3.45 | 0.69 | .36^***^ | .72^***^ | -.21^***^ | .44^***^ |  |  |  |  |  |  |  |  |
| 1. FFMQ-Describe | 3.12 | 0.72 | .50^***^ | .37^***^ | .27^***^ | .73^***^ | .27^***^ |  |  |  |  |  |  |  |
| 1. FFMQ-Acting with awareness | 3.02 | 0.72 | .50^***^ | .15^**^ | .46^***^ | .70^***^ | .03 | .42^***^ |  |  |  |  |  |  |
| 1. FFMQ-Nonjudging | 3.00 | 0.85 | .53^***^ | -.03 | .65^***^ | .67^***^ | -.11^*^ | .29^***^ | .53^***^ |  |  |  |  |  |
| 1. FFMQ-Nonreactivity | 3.07 | 0.57 | .24^***^ | .24^***^ | .06 | .43^***^ | .23^***^ | .20^***^ | -.00 | .13^*^ |  |  |  |  |
| 1. Meta-awareness | 5.38 | 0.95 | .45^***^ | .70^***^ | -.10 | .39^***^ | .54^***^ | .35^***^ | .18^**^ | -.05 | .25^***^ |  |  |  |
| 1. OA | 1.69 | 1.87 | .04 | -.09 | .14^**^ | .09 | -.01 | .01 | .12^*^ | .10 | .06 | -.06 |  |  |
| 1. SA | 3.23 | 1.66 | -.06 | -.10^*^ | .02 | -.06 | -.07 | -.07 | .01 | .00 | -.08 | -.13^**^ | .51^***^ |  |
| 1. Negative emotions | 2.11 | 1.28 | -.11^*^ | -.09 | -.04 | -.15^**^ | -.05 | -.11^*^ | -.06 | -.12^*^ | -.13^*^ | -.11^*^ | .11^*^ | .32^***^ |

Note. PMS = Philadelphia Mindfulness Scale, FFMQ = Five Facet Mindfulness Questionnaire. OA = Objective Ambivalence. SA = Subjective Ambivalence. **p* < .05, ***p* < .01, ****p* < .001

Table S17: Results of regression analyses examining the interaction effects of mindfulness and ambivalence manipulation in predicting SA.

|  |  | Interaction effect | | | |  |
| --- | --- | --- | --- | --- | --- | --- |
|  | Parameter | *b* | *SE* | *t* | *p* |  |
| Model 1 | Condition | 0.939 | 0.166 | 5.653 | .000 |  |
|  | PMS | -0.095 | 0.125 | -0.761 | .447 |  |
|  | Condition x PMS | 0.023 | 0.167 | 0.135 | .892 |  |
| Model 2 | Condition | 0.924 | 0.167 | 5.549 | .000 |  |
|  | PMS-awareness | -0.117 | 0.128 | -0.916 | .360 |  |
|  | PMS-acceptance | -0.046 | 0.123 | -0.372 | .710 |  |
|  | Condition x PMS-awareness | -0.034 | 0.172 | -0.196 | .845 |  |
|  | Condition x PMS-acceptance | 0.074 | 0.171 | 0.432 | .666 |  |
| Model 3 | Condition | 0.931 | 0.176 | 5.744 | .000 |  |
|  | FFMQ | -0.110 | 0.117 | -0.809 | .419 |  |
|  | Condition x FFMQ | -0.301 | 0.176 | -0.144 | .886 |  |
| Model 4 | Condition | 0.937 | 0.168 | 5.580 | .000 |  |
|  | FFMQ-Observe | 0.070 | 0.125 | 0.561 | .575 |  |
|  | FFMQ-Describe | -0.178 | 0.145 | -1.229 | .220 |  |
|  | FFMQ-Acting with awareness | 0.104 | 0.154 | 0.675 | .500 |  |
|  | FFMQ-Nonjudging | -0.028 | 0.137 | -0.204 | .838 |  |
|  | FFMQ-Nonreactivity | -0.085 | 0.121 | -0.698 | .486 |  |
|  | Condition x FFMQ-Observe | -0.277 | 0.183 | -1.514 | .131 |  |
|  | Condition x FFMQ-Describe | 0.245 | 0.199 | 1.231 | .219 |  |
|  | Condition x FFMQ-Acting with awareness | -0.125 | 0.212 | -0.587 | .557 |  |
|  | Condition x FFMQ-Nonjudging | 0.005 | 0.206 | 0.022 | .982 |  |
|  | Condition x FFMQ-Nonreactivity | -0.021 | 0.180 | -0.119 | .905 |  |
| Model 5 | Condition | 0.928 | 0.165 | 5.614 | .000 |  |
|  | Meta-awareness | -0.179 | 0.125 | -1.439 | .151 |  |
|  | Condition x Meta-awareness | -0.002 | 0.167 | -0.010 | .992 |  |
| Model 6 | Condition | 0.933 | 0.166 | 5.619 | .000 |  |
|  | Awareness | -0.202 | 0.159 | -1.270 | .205 |  |
|  | Acceptance | -0.104 | 0.161 | -0.643 | .520 |  |
|  | Condition x Awareness | -0.020 | 0.214 | -0.092 | .927 |  |
|  | Condition x Acceptance | 0.070 | 0.231 | 0.301 | .763 |  |

Note. PMS = Philadelphia Mindfulness Scale, FFMQ = Five Facet Mindfulness Questionnaire. SA = Subjective Ambivalence. Awareness composite is the combination of 4 awareness related subscales (PMS-awareness, FFMQ-observe, FFMQ-describe, Meta-awareness; alpha = 0.929). Acceptance composite is the combination of 3 acceptance related subscales (PMS-acceptance, FFMQ-nonjudging, FFMQ-nonreactivity). All predictors were standardized.

Table S18: Results of regression analyses examining the interaction effects of mindfulness and ambivalence manipulation in predicting negative emotions and associated simple slopes of condition on negative emotions at high and low levels of each mindfulness variable.

|  |  | Interaction effect | | | |  | cond at low mind | |  | cond at high mind | |
| --- | --- | --- | --- | --- | --- | --- | --- | --- | --- | --- | --- |
|  | Parameter | *b* | *SE* | *t* | *p* |  | *b* | *SE* |  | *b* | *SE* |
| Model 1 | Condition | 0.657 | 0.129 | 5.109 | .000 |  |  |  |  |  |  |
|  | PMS | -0.110 | 0.097 | -1.139 | .255 |  |  |  |  |  |  |
|  | Condition x PMS | -0.016 | 0.130 | -0.122 | .903 |  | 0.673*** | 0.183 |  | 0.642*** | 0.182 |
| Model 2 | Condition | 0.653 | 0.128 | 5.086 | .000 |  |  |  |  |  |  |
|  | PMS-awareness | -0.215 | 0.099 | -2.174 | .030 |  |  |  |  |  |  |
|  | PMS-acceptance | 0.006 | 0.095 | 0.059 | .953 |  |  |  |  |  |  |
|  | Condition x PMS-awareness | 0.201 | 0.133 | 1.518 | .130 |  | 0.452* | 0.185 |  | 0.854*** | 0.184 |
|  | Condition x PMS-acceptance | -0.187 | 0.132 | -1.419 | .157 |  | 0.840*** | 0.184 |  | 0.466* | 0.183 |
| Model 3 | Condition | 0.678 | 0.127 | 5.321 | .000 |  |  |  |  |  |  |
|  | FFMQ | -0.146 | 0.094 | -1.562 | .119 |  |  |  |  |  |  |
|  | Condition x FFMQ | -0.103 | 0.128 | -0.803 | .422 |  | 0.780*** | 0.180 |  | 0.575** | 0.181 |
| Model 4 | Condition | 0.689 | 0.128 | 5.379 | .000 |  |  |  |  |  |  |
|  | FFMQ-Observe | -0.109 | 0.095 | -1.147 | .252 |  |  |  |  |  |  |
|  | FFMQ-Describe | -0.085 | 0.111 | -0.766 | .444 |  |  |  |  |  |  |
|  | FFMQ-Acting with awareness | 0.043 | 0.117 | 0.365 | .716 |  |  |  |  |  |  |
|  | FFMQ-Nonjudging | -0.053 | 0.105 | -0.509 | .611 |  |  |  |  |  |  |
|  | FFMQ-Nonreactivity | -0.066 | 0.093 | -0.718 | .473 |  |  |  |  |  |  |
|  | Condition x FFMQ-Observe | 0.210 | 0.140 | 1.506 | .133 |  | 0.478* | 0.189 |  | 0.899*** | 0.190 |
|  | Condition x FFMQ-Describe | 0.059 | 0.152 | 0.391 | .696 |  | 0.629** | 0.199 |  | 0.748*** | 0.198 |
|  | Condition x FFMQ-Acting with awareness | -0.106 | 0.162 | -0.656 | .512 |  | 0.795*** | 0.206 |  | 0.582** | 0.207 |
|  | Condition x FFMQ-Nonjudging | -0.179 | 0.157 | -1.142 | .254 |  | 0.868*** | 0.203 |  | 0.510* | 0.201 |
|  | Condition x FFMQ-Nonreactivity | -0.159 | 0.137 | -1.157 | .248 |  | 0.847*** | 0.186 |  | 0.530** | 0.189 |
| Model 5 | Condition | 0.671 | 0.128 | 5.236 | .000 |  |  |  |  |  |  |
|  | Meta-awareness | -0.208 | 0.095 | -2.191 | .029 |  |  |  |  |  |  |
|  | Condition x Meta-awareness | 0.127 | 0.129 | 0.984 | .326 |  | 0.544** | 0.182 |  | 0.798*** | 0.182 |
| Model 6 | Condition | 0.674 | 0.127 | 5.327 | .000 |  |  |  |  |  |  |
|  | Awareness | -0.291 | 0.121 | -2.396 | .017 |  |  |  |  |  |  |
|  | Acceptance | -0.045 | 0.123 | -0.366 | .714 |  |  |  |  |  |  |
|  | Condition x Awareness | 0.290 | 0.163 | 1.781 | .076 |  | 0.445* | 0.181 |  | 0.904*** | 0.206 |
|  | Condition x Acceptance | -0.415 | 0.176 | -2.352 | .019 |  | 0.973*** | 0.180 |  | 0.376* | 0.179 |

Note. PMS = Philadelphia Mindfulness Scale, FFMQ = Five Facet Mindfulness Questionnaire. SA = Subjective Ambivalence. Awareness composite is the combination of 4 awareness related subscales (PMS-awareness, FFMQ-observe, FFMQ-describe, Meta-awareness). Acceptance composite is the combination of 3 acceptance related subscales (PMS-acceptance, FFMQ-nonjudging, FFMQ-nonreactivity). All predictors were standardized. **p* < .05, ***p* < .01, ****p* < .001.
